# Reliability of qualitative occlusal tooth wear evaluation using an intraoral scanner: A pilot study

**DOI:** 10.1371/journal.pone.0249119

**Published:** 2021-03-25

**Authors:** Renata Travassos da Rosa Moreira Bastos, Priscila Teixeira da Silva, David Normando

**Affiliations:** 1 Post-Graduation Program of Dentistry, Department of Orthodontics, Federal University of Pará, Belém, Pará, Brazil; 2 Dental School, Federal University of Pará, Belém, Pará, Brazil; 3 Department of Orthodontics, Dental School, Federal University of Pará, Belém, Pará, Brazil; University of Zurich, SWITZERLAND

## Abstract

Dental wear analysis through the use of an intraoral scanner is a reality of modern dentistry. This study aimed to investigate the reliability of qualitative tooth wear evaluation through three-dimensional images captured with an intraoral scanner and compared to clinical and photographic examinations. Eighteen adult volunteers of both genders (18 to 55 years old) were submitted to clinical exams, intraoral photographs and intraoral scanning protocol using an optical scanner (TRIOS® Pod, 3Shape, Copenhagen, Denmark). Occlusal tooth wear, from second to second premolars, was measured by two evaluators and reevaluated after 30 days, according to a slight modification of the method described by Mockers et al. Weighted Kappa was used to measure intra and inter-examiner agreement. The Friedman test was used to verify the differences among methods. Random and systematic errors were assessed using Bland-Altman plots. All statistical analysis was performed with p<0.05. There was a substantive agreement for clinical (K = 0.75) and photographic exams (K = 0.79) and a moderate agreement for intraoral scanner analysis (K = 0.60) for inter-examiner evaluation. A substantial intra-examiner agreement was obtained for both evaluators. No significant difference between the methods was observed (p = 0.7343 for examiner 1 and 0.8007 for examiner 2). The Bland-Altman plot confirmed no systematic errors between the methods and a random error of 0.25 with the scanner method when compared to clinical assessment. All three methods showed reliability in qualitative occlusal tooth wear evaluation. Intraoral scanning seems to be a sound and reliable tool to evaluate tooth wear when compared to traditional methods, considering the lower inter-examiner agreement and the inherent limitations of this pilot study. Further research will be necessary in order to achieve more robust evidence.

## Introduction

Tooth wear refers to diverse conditions that cause loss of mineralized tooth tissue without bacterial action or dental trauma [1]. Although these conditions have common characteristics, they show etiological and morphological differences, that are related to mechanical tooth-to-tooth contact on the occlusal surfaces, to tooth contact with food during the chewing process or no masticatory movements, and to the chemical dissolution of enamel through acid from diet or gastric sources [1,2]. This mechanism is associated with different non-carious tooth tissue loss, namely attrition, abrasion and erosion [3].

Erosion is the gradual and irreversible loss of dental hard tissue due to non-bacterial acidic agents [4,5], and are mostly related to diet, medication, vomiting, and reflux [5,6]. There is concern with the increased prevalence of dental erosion among children and adolescents [7,8]. Attrition is the physiological wearing through tooth-to-tooth contact without the presence of food or foreign objects [9]. It is related to parafunctions and clinically characterized by wear facets [10]. Abrasion is due to the friction of teeth with exogenous objects or abrasive substances [1]. Finally, abfraction was added to this group of disorders causing dental hard tissue loss and it is characterized by microfractures and microstructural loss that culminates in cervical lesions, caused by a force application point outside the long tooth axis [11].

Tooth wear has been commonly related to the aging process and to the type of diet typically associated with remote indigenous populations, groups that had little or no interaction with modern society [12], as well as increasing levels of tooth wear are significantly related to aging processes in general populations, as it was observed in a previously published systematic review [13]. Additionally, excessive wear can cause dental sensitivity, irreversible pulpal damage, loss of vertical dimension of occlusion, and damage to facial aesthetics, the stomatognathic system and the dental organ [14].

Conventional impressions and study casts can be used to evaluate dental wear. However, alginate and gypsum material casts are not designed to assess initial stages of tooth wear, as it is difficult to reproduce details of the dental surfaces, especially dental erosion [15]. Other methods described in the literature are the conventional clinical examination and intraoral photographs. In both cases, tooth wear can be measured with scores that correspond to a level of wear [12,16,17]. Intraoral photographs are associated with a lack of sensitivity to detect small changes and loss of tooth substances [8]. It can be difficult to diagnose the various forms of tooth wear in the clinical examination because of the morphological variations, with the most common feature being a reduction in the length of the clinical crown [18,19]. In addition, it is easier to perform a qualitative analysis of categorical data. However, the quantitative assessments tend to be more sensitive and more sophisticated equipment should be used when performing quantitative assessment [20].

The ability to analyze dental wear through the use of an intraoral scanner, which captures and generates three-dimensional (3D) images, is a reality of modern dentistry [21]. The analysis of these images allows for not only the possibility of storage, but also for the quantification, through longitudinal studies, of the volume of dental tissue lost through wear [22]. Therefore, this study aims to evaluate the reliability of qualitative tooth wear of occlusal surfaces obtained through 3D images captured with an intraoral scanner when compared to conventional methods of clinical examination and photographic analysis, through categorical data. The null hypothesis of the study was that there would not be a difference in tooth wear evaluation between intraoral scanner and the other two conventional methods, clinical examination and intraoral photographs.

## Materials and methods

This study was submitted to the Ethics Research Committee of the Institute of Health Sciences, Federal University of Pará (ICS/UFPA) and was approved under the protocol number 1.996.860/2017 (S1 File). In accordance with the Resolution 466/12 of the National Health Council (NHC), the individuals who voluntarily agreed to participate in the research signed a free and informed consent form, authorizing the clinical examination, intraoral photographs and intraoral scanning, as well as the use, storage and publication of the collected data for this study and other scientific research purposes.

The sample size (*n*) necessary to identify a predicted intraclass correlation coefficient (ICC) of 0.8 with a lower confidence limit of 0.6 (*r*), 80 per cent power, and alpha level of 5 per cent was 16 individuals [23,24]. Both calculations were performed using BioEstat software (version 5.3, Mamirauá Maintainable Development. Institute, Belém, Pará, Brazil). Considering a sample loss of around 10%, eighteen adult individuals (10 men and 8 women) with ages ranging from 18 to 55 years old were recruited. The following exclusion criteria were adopted: individuals missing four or more permanent teeth and undergoing current orthodontic treatment. Each participant was evaluated for dental wear using all the three methods previously mentioned.

Tooth wear was evaluated using a slightly modified version [25] of the classification system published by Mockers et al. [16]. The occlusal surfaces of the first and second premolars, canines, central and lateral incisors of the upper and lower arches were examined. Then the following scores were recorded for each tooth, depending on its level of tooth wear: 0 = no wear; 1 = only enamel wear; 2 = dentin wear in which the occlusal/incisal surface has more enamel than dentin; 3 = dentin wear in which the occlusal/incisal surface has more dentin than enamel; and 4 = advanced wear, near or beyond the pulp. This evaluation method was used in all three techniques of dental wear assessment: clinical examination, intraoral photographs and intraoral scanning.

The calibration method between the two examiners was performed by the evaluation of the occlusal surfaces of 20 teeth (premolars, canines and incisors) from four patients, 80 teeth in total, using the same criteria for all methods. These results were analyzed through the weighted Kappa test in order to measure their inter-examiner agreement for each method.

### Clinical evaluation

Two previously calibrated evaluators clinically examined the patients, a graduate student in dentistry (PTS) and a specialist in Orthodontics (RTRMB). The clinical evaluations were performed with the aid of a clinical mirror and with light from the dental team’s reflector. The aim was to analyze the patterns and levels of wear present in the occlusal surfaces of the participants’ teeth on the upper and lower arches, recording the scores assigned to each evaluated tooth using the same slight modification [25] of the classification method described by Mockers et al. [16].

### Occlusal intraoral photographs

**Fig 1:**
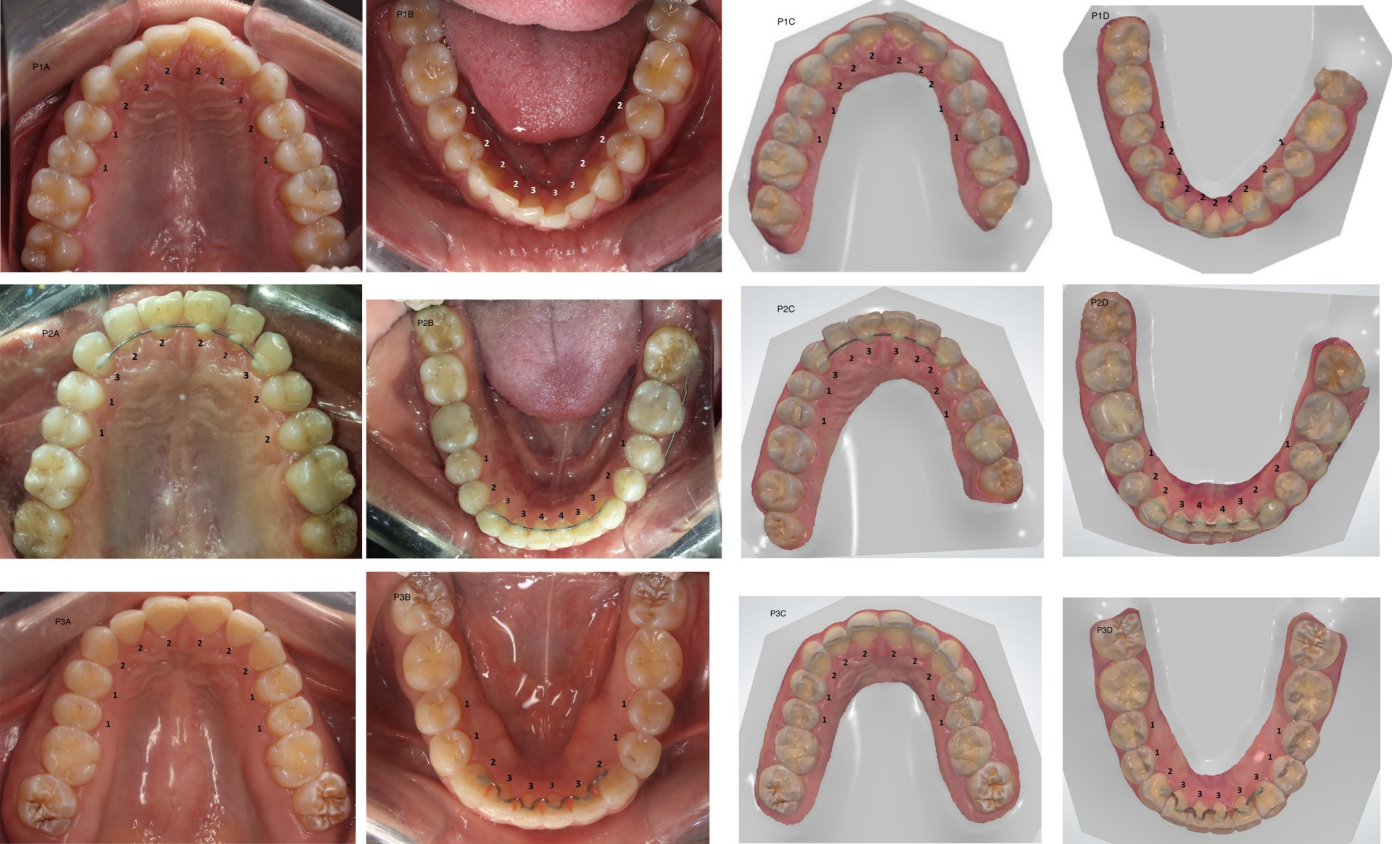
Patient 1 (P1), patient 2 (P2), and patient 3 (P3) occlusal intraoral photographs and 3D images captured with the intraoral scanner of a modified tooth wear measurement performed by the same operator. A- upper dental arch photo; B- lower dental arch photo; C- upper dental arch scan; D- lower dental arch scan. Scores: 0- absence of wear; 1- enamel wear only; 2- dentin wear, with the occlusal/incisal face showing more enamel than dentin; 3- dentin wear, with the occlusal/incisal face showing more dentin than enamel; 4- advanced wear stage, near or beyond the pulp.

A standardized occlusal photograph was obtained for each participant, always by the same two operators, one to take the photo and the other to ensure the correct placement of the accessories. The teeth were blow dried and intraoral occlusal photographs were taken of the upper and lower arches using a digital camera with a resolution of 18 megapixels (Rebel T3i, Canon, Tokyo, Japan) from the shortest distance to the object capable of generating the image focus. An occlusal mirror was used with a light (Osung, Osung CO MND, Chicago, USA) and lip retractors (Fig 1). The mirror was placed in contact with the opposite dental arch to be photographed and the camera was positioned as perpendicularly as possible to the mirror. After the photographs were taken, they were exported to an Apple MacBook laptop (Apple Inc., Cupertino, USA) and were evaluated. The images were blinded. The dental wear scores of the occlusal surface of each evaluated tooth of the upper and lower arches were recorded following the same classification system [16], by the two calibrated examiners.

### Intraoral scanning

A standardized scan of the upper and lower arches was obtained for each volunteer by the same operator. Every day, before the start of activities, the intraoral scanner was calibrated, always following the manufacturers’ recommendation for all the stages of the scanning process. Then, the patient was positioned in the dental chair and a light intraoral scanner (TRIOS^®^ Pod, 3Shape, Copenhagen, Denmark) was used to obtain images of both arches. The scanner tip was positioned as close as possible to the teeth. The operator started the scan on the occlusal surface, continuing through the palatal/lingual surface and, finally, through the buccal area of the teeth of the upper and lower arches, respectively. The 3D colored images obtained from the intraoral scanner were exported to the OrthoAnalyzer^TM^ 3D software (OrthoAnalyzer Orthodontics, 3Shape Medical A/S, Copenhagen, DK) on an Apple MacBook laptop (Apple Inc., Cupertino, USA) (Fig 1). The colored images of the occlusal surfaces of the selected teeth were evaluated and the level of dental wear was assigned by categorical data as described in the previous techniques [16]. The images were blinded. The two calibrated examiners performed the evaluations.

### Statistical analysis

Weighted Kappa was used to evaluate the intra-rater agreement for each method through a comparative analysis of two measurements, after a 30-day interval, by the same examiners. Weighted Kappa was also used to evaluate inter-rater agreement for each method. For intra and inter-examiner agreement, the weighted Kappa was used in a perspective of agreement per tooth, taking into account the scores recorded for each tooth, and not per individual. The Friedman test was used to verify the differences among the three methods and was performed by selecting elements from the same hemiarch for evaluation: the lower-right central incisor, lateral incisor and canine (LR1, LR2 and LR3). For this specific analysis, it was considered the first assessment of both examiners, since the clinical examination was not reassessed after 30 days. To evaluate systematic error (bias) and random error (precision), a Bland-Altman analysis was applied using the arithmetic mean for each individual obtained from all the scores of their teeth. Statistical analysis was performed using BioEstat (version 5.3, Mamirauá Institute, Belém, Brazil), MedCalc (MedCalc Software bvba, Ostend, Belgium) and VassarStats (VassarStats website, Vassar College, Poughkeepsie, USA), with a significance level of p<0.05.

## Results

The weighted Kappa test was used to verify the intra-examiner agreement between the evaluations performed at time 1 and at time 2, after a 30-day interval. The results showed substantial agreement, with evaluator 1 obtaining Kappa values of K = 0.663 and K = 0.661 for the photography and the scanner, respectively, and evaluator 2 K = 0.684 for photography and K = 0.641 for the scanner analysis (Table 1). The reevaluations, performed at time 2, only used previously collected images, so the clinical examination method has no reevaluation data since the participants should be recruited again for evaluation and the amount of dental wear could change after 30 days, even if minimally, which may interfere with the result of the weighted Kappa for intra-examiner agreement. The results were also analyzed through the weighted Kappa test in order to measure their inter-examiner agreement for each method. There was a substantive agreement for clinical (K = 0.746) and photographic exams (K = 0.791) and a moderate agreement for intraoral scanner analysis (K = 0.595) (Table 1). The results showed that all three methods, when performed by previously calibrated evaluators, were reliable. There was no significant difference among them for each evaluator (p = 0.7343 for examiner 1 and p = 0.8007 for examiner 2) (Table 2).

**Table 1:** Intra and inter-examiner agreement values obtained through weighted Kappa test.

	Weighted Kappa inter-examiner calibration	Agreement analysis	
		Weighted Kappa T1 x T2: Examiner 1	Weighted Kappa T1 x T2: Examiner 2
**Clinical Exam**	0.746	----	----
**Intraoral Photographs**	0.791	0.663	0.684
**Intraoral Scanner**	0.595	0.661	0.641

T1 and T2 means time 1 and time 2.

**Table 2:** Median and standard deviation values for the Friedman test.

		Friedman test		
		Median	Standard Deviation	p-value
**Examiner 1**	Clinical Exam	2	0.6212	0.7343
	Photography	2	0.6007	
	Scanner	2	0.6727	
**Examiner 2**	Clinical Exam	2	0.6742	0.8007
	Photography	2	0.6553	
	Scanner	2	0.6553	

**Fig 2:**
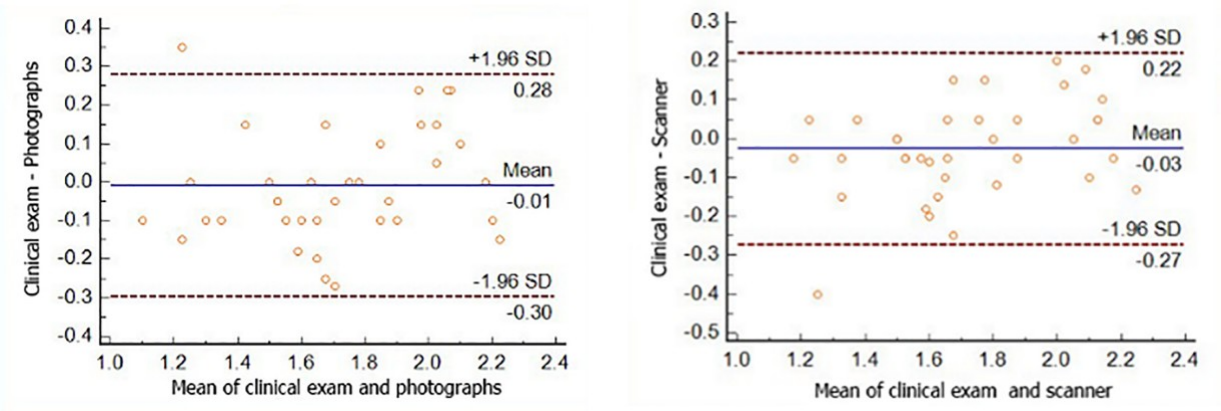
Bland-Altman test comparison between A- the clinical exam and intraoral photography and B- the clinical exam and intraoral scanner. ** From the visualization of the dispersion graph, displays low variability and a significant concordance between the methods, with no large dispersions or outliers**.

Inter-method systematic and random errors were confirmed using a Bland-Altman scatter plot. Because this plot is used to graph only two methods, data from the clinical examination was used as the standard for comparison with the others. Results were pairwise compared with those of the photographic and scanning methods, using both evaluators’ mean values for each individual. The results obtained from the other methods showed little individual variability in relation to those from the clinical examination, and there were no large dispersions or outliers. As the mean line of the two methods is on the zero line, this confirmed no significant systematic errors between the methods for the analysis of tooth wear. In addition, it can be inferred that the random error (precision) was <0.3 for photographs or intraoral scanner when compared to the standard method (Fig 2).

## Discussion

This study evaluated the reliability of qualitative occlusal tooth wear assessment using an optical intraoral scanner, proving that this new approach is effective in performing the proposed objectives and is a reliable method. Additionally, the null hypothesis was proven to be true, which indicates that all three methods are capable of evaluating the amount of occlusal tooth wear through a slight modification in the categorical index proposed by Mockers et al. [16].

Inter-examiner assessments showed a substantial agreement between the measures obtained at time 1 and at time 2, with minimal variation in the Kappa value for both evaluators. Thus, the subsequent analysis of tooth wear after a period of time produces similar results to those obtained in the initial evaluation for photography and scanning, and does not influence the examiners’ accuracy, as they had been previously calibrated. This evaluation was not possible for clinical visual analysis since the 30-day time interval used between the two measurements could interfere with the amount of wear evaluated. Once there was intra and inter-examiner agreement when using the scores recorded for each tooth, the diagnostic assessment method becomes reliable and can be applied to the evaluation of dental wear of each individual patient in clinical practice.

Tooth wear, in addition to being associated with the consumption of unprocessed or abrasive foods [12], is present in a large part of the adult and younger population because it is strongly related to parafunctional habits, dental disorders [26,27] or even orthodontic movement [22]. Furthermore, tooth wear has been used to evaluate the aging process [12], diet habits [25] and acculturation process [28] among remote indigenous populations.

There are several well accepted scoring systems for assessing and monitoring occlusal tooth wear published in the literature, both qualitative and quantitative [27]. Some of these indices take into account wear sites due to dental erosion [29,30], in which the lesions are evaluated on the buccal, occlusal and lingual aspects of the teeth [31]. Other classifications, in turn, categorize tooth abrasion using dental enamel wear facets [32,33]. Finally, some anthropological classifications allow the quantification of tooth wear in ancient populations [34,35], where tooth wear can be considered a direct evidence of what the individual ate in the past, and may be an indicator of the acculturation process in remote populations [28].

The evaluation of tooth wear is usually performed through a traditional clinical examination to measure the levels of wear of various dental structures, such as the enamel, dentin and pulp. Previous studies that assessed dental wear have used clinical examination, a fast, simple, easy and replicable method to be performed by well-calibrated examiners [12,16,25,36]. Although our findings have confirmed the accuracy of tooth wear examination through clinical assessment, this methodology makes it difficult to compare the results longitudinally.

Our results show that high-quality intraoral photographs also proved to be a reliable method for assessing dental wear, producing substantial agreement with respect to the clinical examination results (Fig 2). The Bland-Altman plot confirmed no systematic error between the methods and a random error of 0.3 with intraoral photography method when compared to visual clinical assessment. Once it is not possible to manipulate such photographs, certain details are lost due to the difficulty of reaching some parts of the mouth and because of unwanted shadows [37]. This methodology is actually used within a clinical practice setting to monitor tooth wear in many patients, with different etiologies [8,18]. However, these methods have some inherent limitations that may compromise the accurate assessment of tooth wear measurements [18,19], and among them, four important variables greatly affect the quality of intraoral photographs. These include training in photography, both for the dentist and the dental staff, experience in taking photographs, time taken to obtain the photos, and characteristics of the camera, accessories and equipment [37]. Some errors related to the process of taking a photograph include camera failure positioning, poor focusing, and over- or underexposure. Errors related to intraoral photographs include excessive saliva bubbles, a fogged mirror, dark buccal corridors, failure in the tongue and retractor positions, and darkening and hindering the field of view [38].

In the search for innovative technologies that can produce more efficient clinical and scientific results, the intraoral scanner shows an advantage at capturing highly realistic 3D images. This method allows the operator to generate and manipulate the image as soon as it is obtained, eliminates the steps of cleaning, disinfecting, and casting used in the traditional alginate impressions [39], presents the possibility of obtaining reliable measurements of the dental arches [21,40] and offers better performance than ordinary photography in terms of image capture, especially in areas considered difficult to reach (Fig 1), like the distal aspects of the molars [37]. Additionally, the shortest time spent on the laboratory steps is an advantage since it is possible to immediately send the file model obtained through scanning [21]. However, the specific loss of some tooth details results in the lowest level of inter-examiner agreement on the amount of tooth wear evaluated (K = 0.595). This can be explained by the digital method employed, and the accuracy of the generated digital model images, which is part of the limited resolution of the scanner [21].

Substantial agreement of the intraoral scanning method has been observed when compared with the clinical examination with a sound replicability (Fig 2). The Bland-Altman plot confirmed no systematic error between the methods and a random error of 0.25 with the scanner method when compared to the clinical assessment. The possibility of manipulating the image along various axes with intraoral scanning also helps in the evaluation of tooth wear in this study, because it was ideal for visualizing small facets perceived through changes of light in the occlusal-incisal region when moving the image. The scanning method also has the advantages of allowing for easy storage of high-quality images and exporting of data, thus creating the possibility of analyzing digital models for treatment planning, evaluation and the creation of a database for scientific research [41–43]. Furthermore, the 3D images permit a longitudinal quantification of the volume of dental tissue lost through wear [22].

The high cost of scanners and major chairside time required to take the digital images with intraoral scanners are important factors to be considered [40,44]. The acquisition of new technologies allows for faster and more efficient 3D image capturing procedures [44] that associated with more compact devices can provide greater comfort to the patient [40]. Contradictorily, a previously published study that compares conventional and digital impressions using a new generation powder-free scanner showed that the scanner is more time efficient than the traditional impression and more comfortable for patients [21]. Additionally, the high cost to purchase and maintain the equipment hinders routine clinical use [44]. However, the advantages of the digital flow with the development of new technologies and updated versions will encourage greater use of the intraoral scanner for many clinical applications [21,40,44].

Some inherent characteristics of the study design were important limitations of this research. Although evidence has shown that age is one of the most important factors that influence the amount of tooth wear [12,13], a heterogeneous sample and a large age range would not strongly affected the dental wear assessment, since tooth wear is a poor estimator of chronological age in the urban population [12]. Considering this scenario, the age and the consequent ease or difficulty in assessing tooth wear should not substantially impact the reliability of the method. Additionally, the presence of dental crowding would not be able to hinder the analysis of wear, since only the occlusal surface of the teeth was evaluated. Another restriction was that the assessment of other tooth surfaces was not considered in the study design. Regarding orthodontic treatment, only cases of current orthodontic treatment were considered as exclusion criteria, since it is difficult to scan teeth with metallic appliances. However, it is well known that orthodontic treatment may aggravate tooth wear [45,46], and even patients with previous treatment should have been excluded, as their tooth wear would be more easily detectable. In summary, the results of this pilot study were promising, but the amount of wear prior to the study should have been considered as an inclusion or exclusion criterion, as if there is some tooth wear that is negligible but still detectable with naked eye, it will be more difficult to be pointed out when using 3D images. The goal of the study is the reliability of the evaluation methods, mainly intraoral scanner, and it depends on the degree of dental wear presented by the patient.

A major problem of the study is related to the use of a very brief categorical index for tooth wear assessment. By definition and by the scientific evidence available, the use of this qualitative index considerably reduces the precision and reproducibility of each tooth wear assessment method evaluated in the study. The applied methodology, including the statistical approach, should be considered with caution, since a diagnostic assessment method is tested. These such index cannot be named a gold standard, even if such assessment methods are widely used, as it was done with clinical examination in Bland-Altman analysis. The study tests the use of an intraoral scanner, which is a relatively new, but very powerful tool, added to the contemporary armamentarium of dental practitioners. Intraoral scans offer great possibilities for very accurate tooth wear assessment using 3D superimposition techniques [47].

This study compared three methods to verify their reliability in the quantification of tooth wear. The intraoral scanner proved to be reliable for this purpose, as it met the objectives applied in this study. The method also demonstrated substantial agreement and precision when compared to direct measurements of the standard methods.

## Conclusions

According to this pilot study and its inherent limitations, intraoral scanning seems to be a sound and reliable tool to evaluate tooth wear for clinical and scientific purposes when compared to traditional methods, considering the lower inter-examiner agreement and the limitations in the study design. Further research will be necessary in order to achieve more robust evidence.

## Supporting information

S1 File Approval of the Ethics Research Committee of the Institute of Health Sciences, Federal University of Pará (ICS/UFPA). (PDF)

S2 File Statistical analysis. (DOCX)

S3 File Raw data. (XLSX)
