# Supplementary material for: Reliability of qualitative occlusal tooth wear evaluation using an intraoral scanner: A pilot study
Source: PLoS One. 2021 Mar 25;16(3):e0249119. doi: 10.1371/journal.pone.0249119 (PMC7993778; doi:10.1371/journal.pone.0249119)
Supplement: S1 File — (PDF) [file pone.0249119.s001.pdf]

PARECER CONSUBSTANCIADO DO CEP

DADOS DO PROJETO DE PESQUISA

Título da Pesquisa: Avaliação de desgaste dentário através de exame clínico, fotografias e escaneamento intraoral.

Pesquisador: Antonio David Corrêa Normando

Área Temática:

Versão: 1

CAAE: 63710916.5.0000.0018

Instituição Proponente: Instituto de Ciências da Saúde da Universidade Federal do Pará - ICS/ UFPA

Patrocinador Principal: Financiamento Próprio

DADOS DO PARECER

Número do Parecer: 1.996.860

Apresentação do Projeto:

O objetivo deste estudo é avaliar a presença e o nível de desgaste dentário, através de exame clínico convencional, imagens bidimensionais de fotografias intraorais e imagens tridimensionais geradas por um scanner intraoral. Para estimar o tamanho da amostra, será realizado um cálculo amostral. Serão selecionados indivíduos adultos, com idade entre 18 e 50 anos, de ambos os sexos. Os pacientes serão avaliados clinicamente buscando analisar o grau de desgaste presente nas superfícies oclusais de 2º pré-molar a 2º pré-molar dos arcos superior e inferior. O desgaste dentário também será avaliado nas imagens fotográficas intrabucais de todos os pacientes, assim como nas imagens geradas pelo scanner intraoral.A avaliação da variável nas imagens obtidas por fotografias e escaneamento intraoral serão repetidas após 30 dias. A estatística não-paramétrica de

Friedman será empregada para verificar a diferença entre as amostras. O erro sistemático no exame do desgaste dentário será verificado através da análise comparativa das duas mensurações executadas nos diferentes tempos empregando-se, para isso, o teste Kappa Ponderado. Para avaliação da concordância entre os três métodos de avaliação do desgaste dentário, será aplicado o teste de Bland-Altman.

Objetivo da Pesquisa:

Objetivo Primário:

Avaliar a presença e o nível de desgaste dentário, através de exame clínico convencional, imagens bidimensionais de fotografias intraorais e imagens tridimensionais geradas por um scanner intraoral.

Objetivo Secundário:

1- Verificar se a avaliação de desgaste dental realizada por três métodos distintos: faz-se confiável em todos. 2- Comparar a replicabilidade e precisão dos métodos de avaliação do desgaste dentário, objetivando a validação do scanner intraoral como um novo método para avaliação desta desordem.3- Analisar e comparar o tempo necessário para a realização de cada um dos métodos de avaliação utilizados no estudo.

Avaliação dos Riscos e Benefícios:

Riscos:

Os riscos decorrentes da participação na pesquisa são a quebra de sigilo das informações relativas aos participantes, caso estes dados sejam furtados. Nenhum risco de caráter psicológico.

Benefícios:

Os benefícios aos participantes da pesquisa serão a sua contribuição para a avaliação da confiabilidade e replicabilidade dos métodos de análise de desgaste dental, e na possível validação de um novo método para avaliação desta desordem bucal que, atualmente, atinge grande parte da população.

Comentários e Considerações sobre a Pesquisa:

O protocolo apresentado dispõe de metodologia e critérios definidos conforme resolução 466/12 do CNS/MS.

Considerações sobre os Termos de apresentação obrigatória:

Os termos apresentados contemplam os sugeridos pelo sistema CEP/CONEP.

Conclusões ou Pendências e Lista de Inadequações:

Diante do exposto somos pela aprovação do protocolo. Este é nosso parecer, SMJ.

Considerações Finais a critério do CEP:

Este parecer foi elaborado baseado nos documentos abaixo relacionados:

Tipo Documento	Arquivo	Postagem	Autor	Situação

Endereço: Rua Augusto Corrêa nº 01-Sl do ICS 13 - 2º and.
Bairro: Campus Universitário do Guamá **CEP:** 66.075-110
UF: PA **Município:** BELEM
Telefone: (91)3201-7735 **Fax:** (91)3201-8028 **E-mail:** cepccs@ufpa.br

Informações Básicas do Projeto	PB_INFORMAÇÕES_BÁSICAS_DO_PROJETO_626604.pdf	09/01/2017 18:53:37		Aceito
Projeto Detalhado / Brochura Investigador	projeto_desgaste_dentario.docx	09/01/2017 18:52:17	Antonio David Corrêa Normando	Aceito
Declaração de Pesquisadores	termo_compromisso_pesquisador.docx	09/01/2017 18:49:53	Antonio David Corrêa Normando	Aceito
Outros	onus_financeiro.docx	29/10/2016 21:34:48	Antonio David Corrêa Normando	Aceito
TCLE / Termos de Assentimento / Justificativa de Ausência	TCLE.docx	27/06/2016 19:05:59	Antonio David Corrêa Normando	Aceito
Declaração de Pesquisadores	termo_aceite_orientador.docx	27/06/2016 18:37:44	Antonio David Corrêa Normando	Aceito
Declaração de Pesquisadores	carta_encaminhamento_cep.docx	27/06/2016 18:37:14	Antonio David Corrêa Normando	Aceito
Declaração de Instituição e Infraestrutura	termo_consentimento_instituicao.docx	27/06/2016 18:34:51	Antonio David Corrêa Normando	Aceito
Folha de Rosto	folha_rosto.docx	27/06/2016 18:29:26	Antonio David Corrêa Normando	Aceito

Situação do Parecer:
Aprovado

Necessita Apreciação da CONEP:
Não

BELEM, 03 de Abril de 2017

Assinado por:
Wallace Raimundo Araujo dos Santos
(Coordenador)
