# Supplementary material for: Reliability of qualitative occlusal tooth wear evaluation using an intraoral scanner: A pilot study
Source: PLoS One. 2021 Mar 25;16(3):e0249119. doi: 10.1371/journal.pone.0249119 (PMC7993778; doi:10.1371/journal.pone.0249119)

Statistical analysis

Clinical exam – Examiner 1


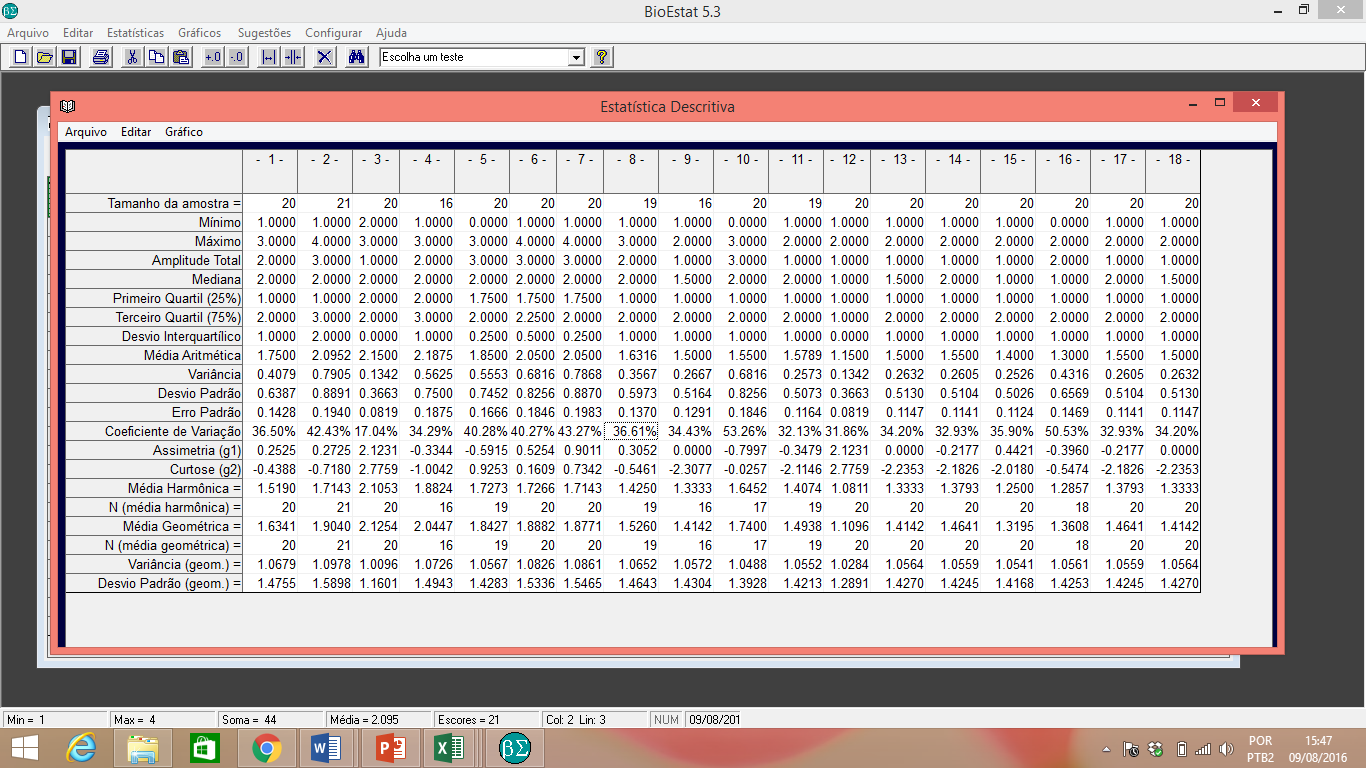


Clinical exam – Examiner 2


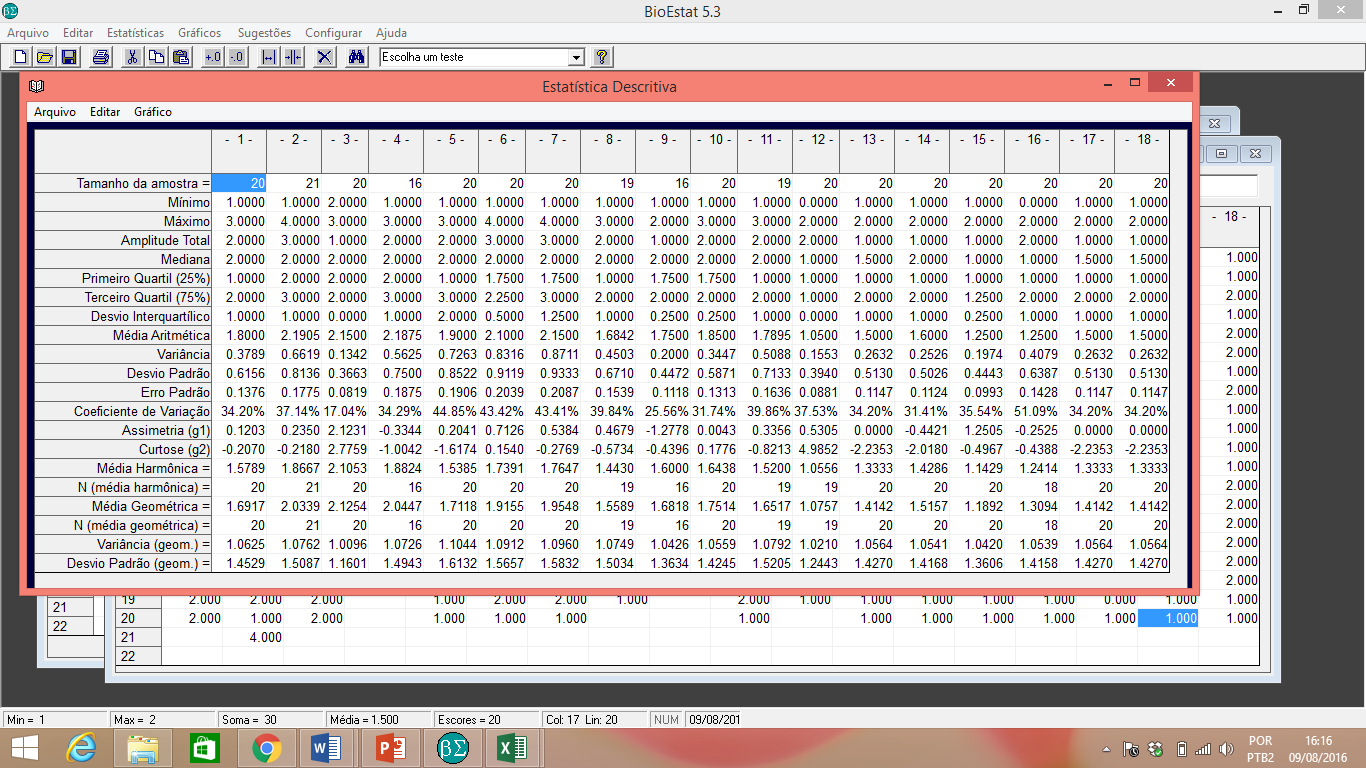


Scanner Examiner 1


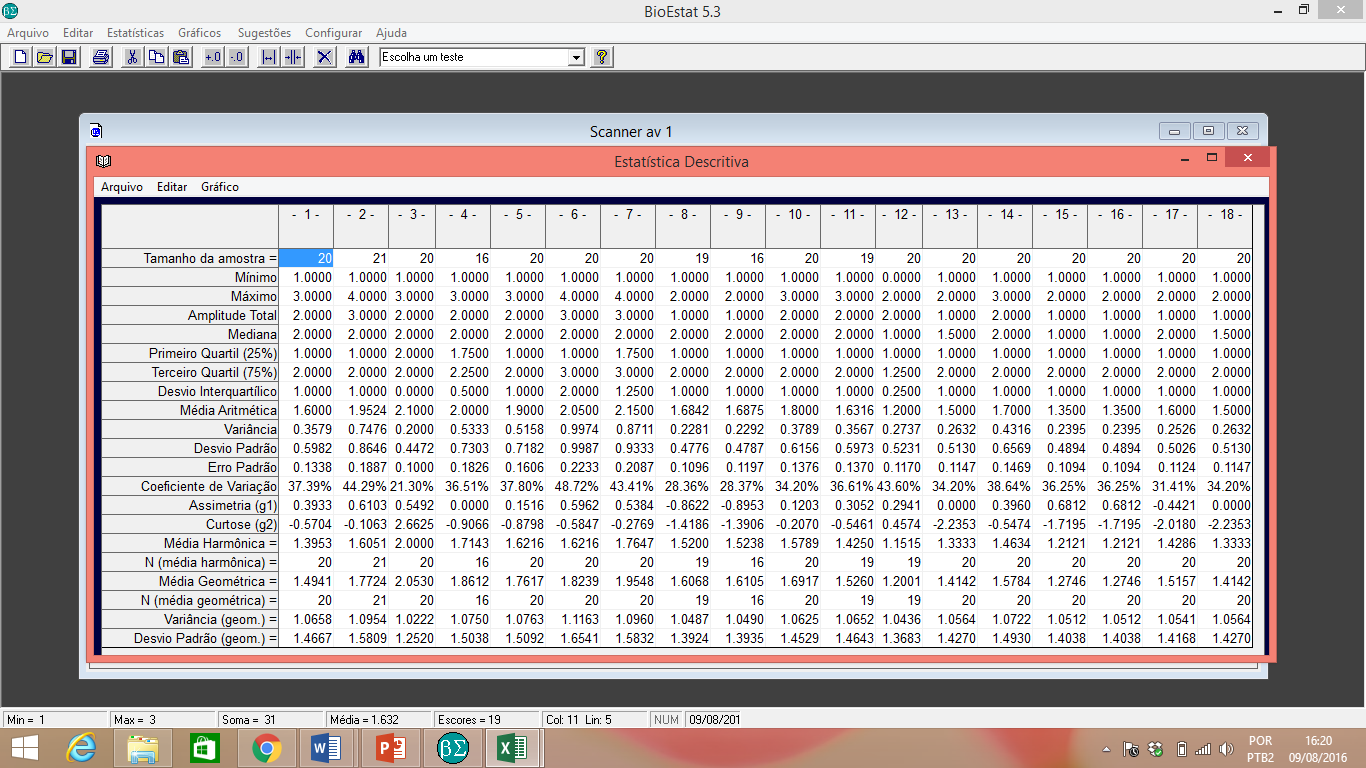


Scanner Examiner 2


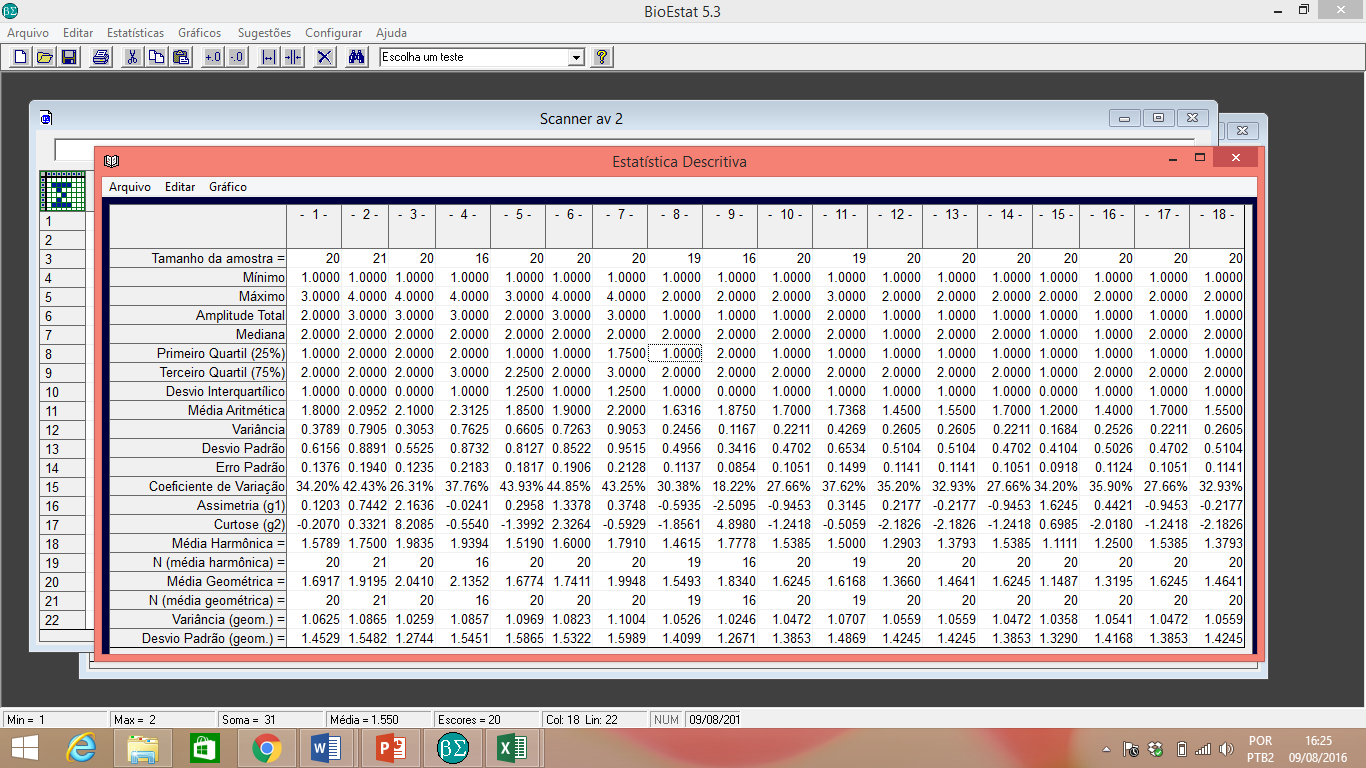


Photo Examiner 1


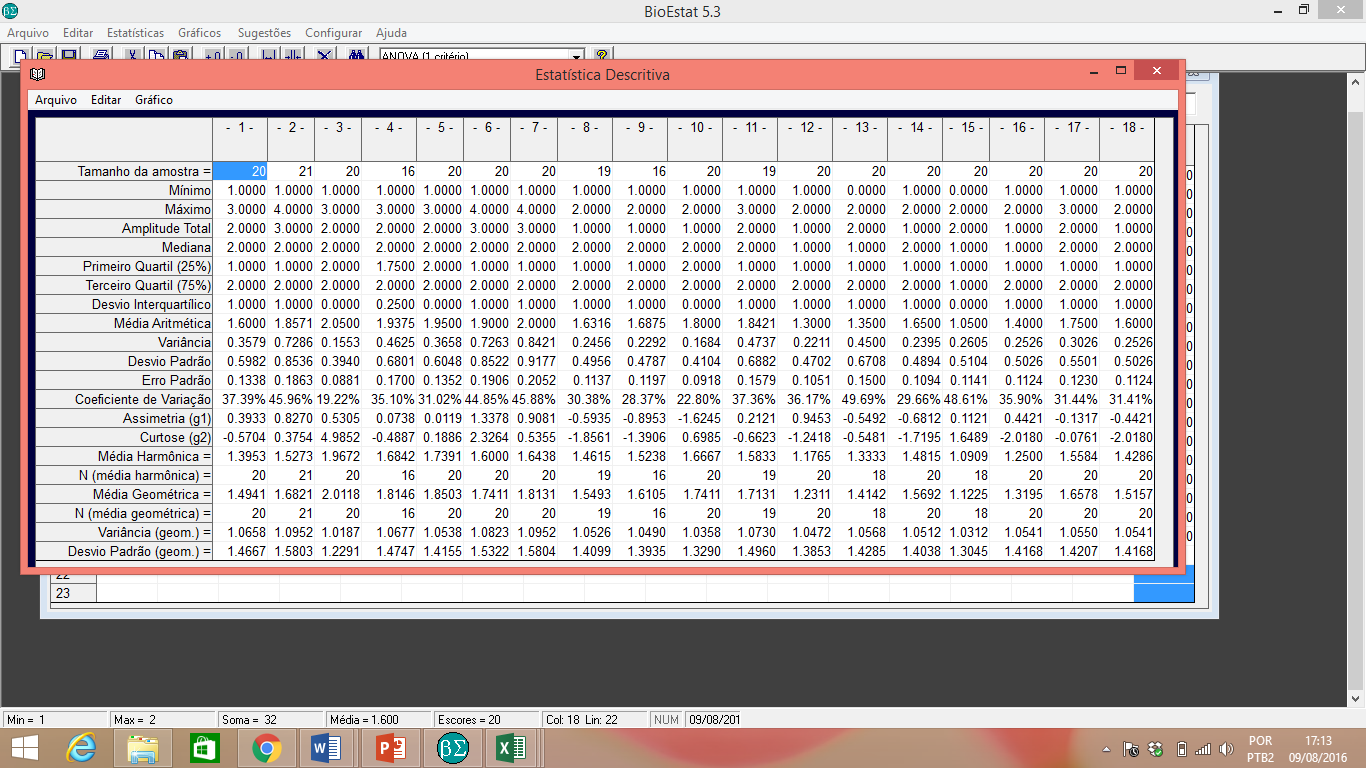


Photo Examiner 2


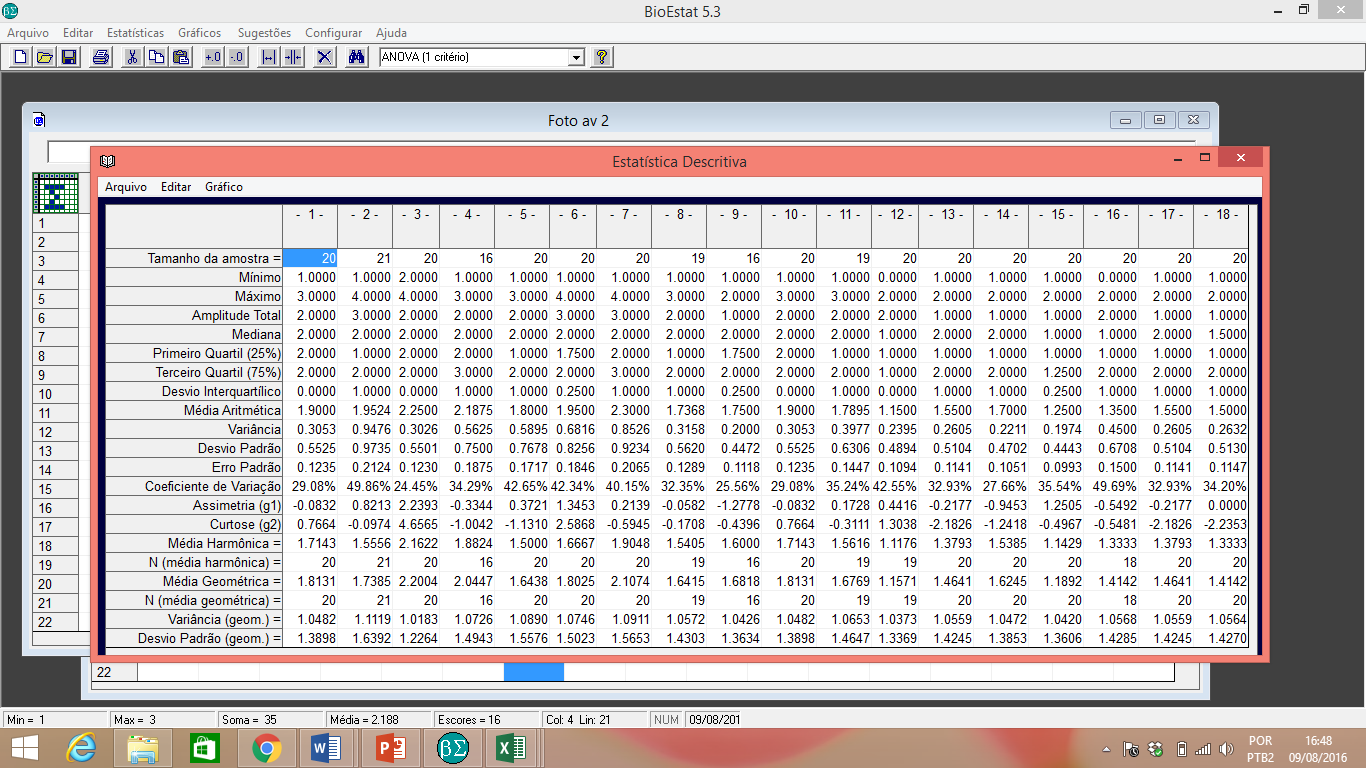


Weighted Kappa

Clinical exam


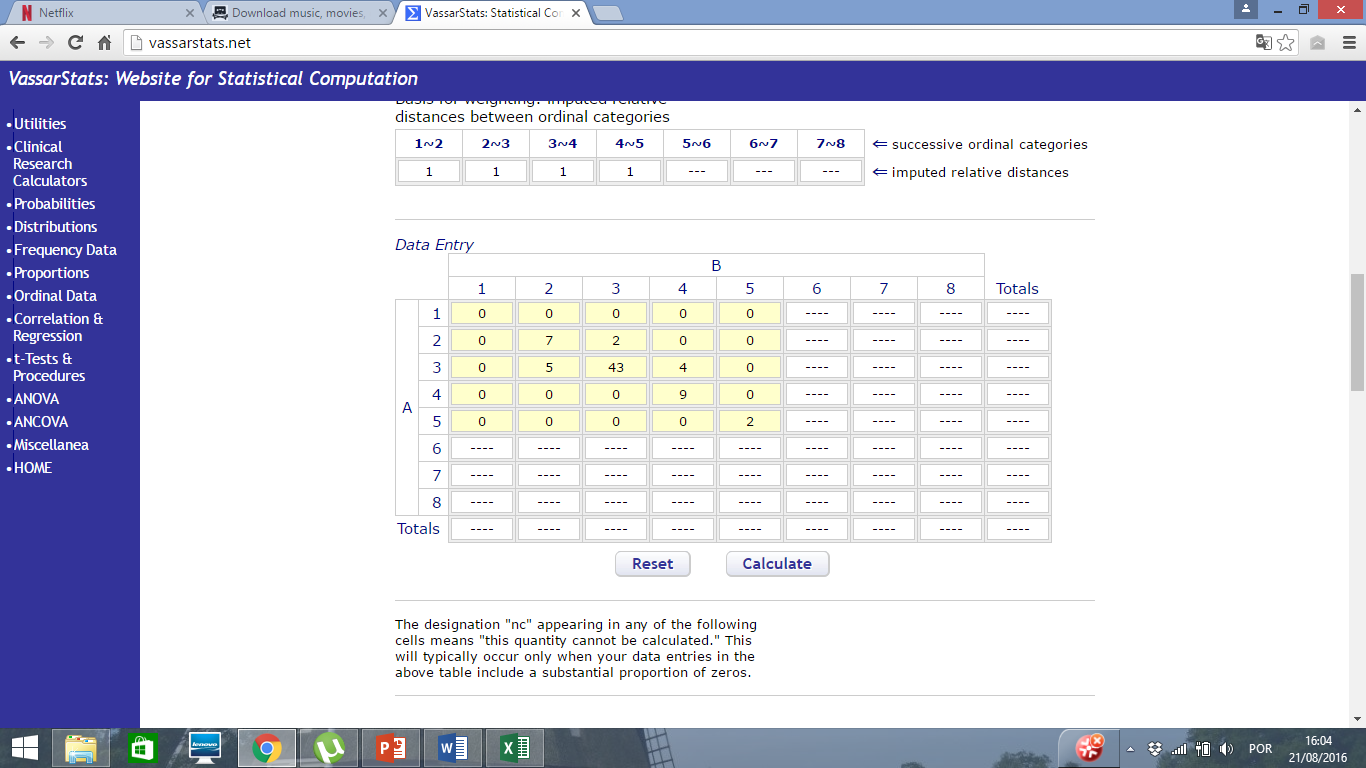


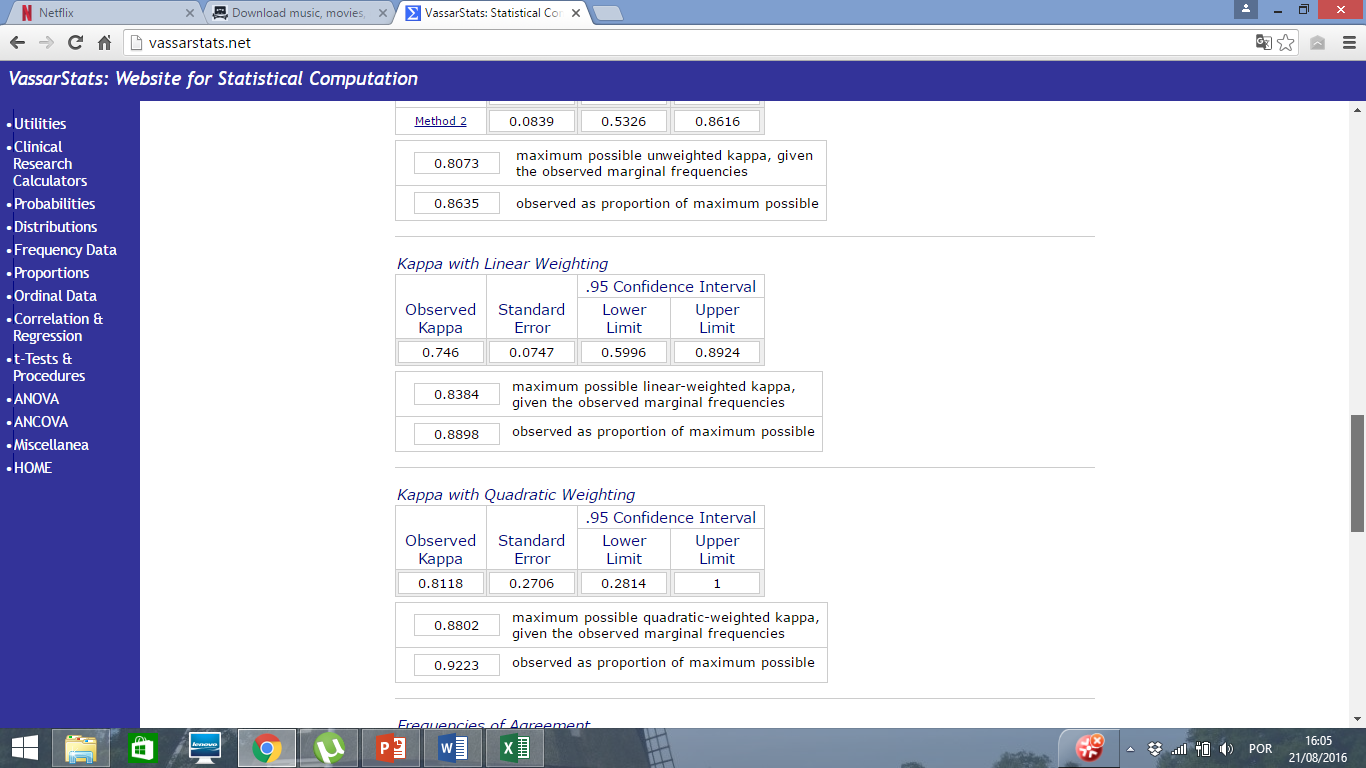


Photo


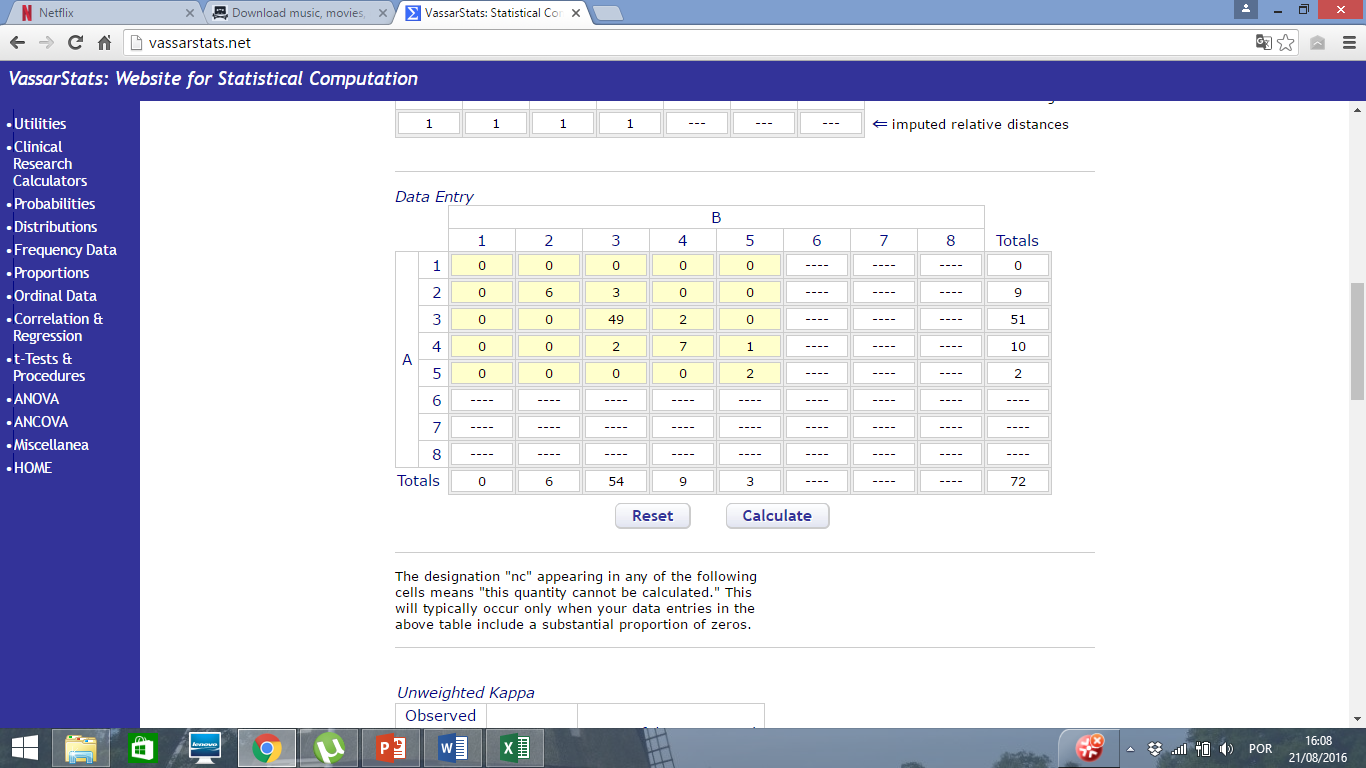


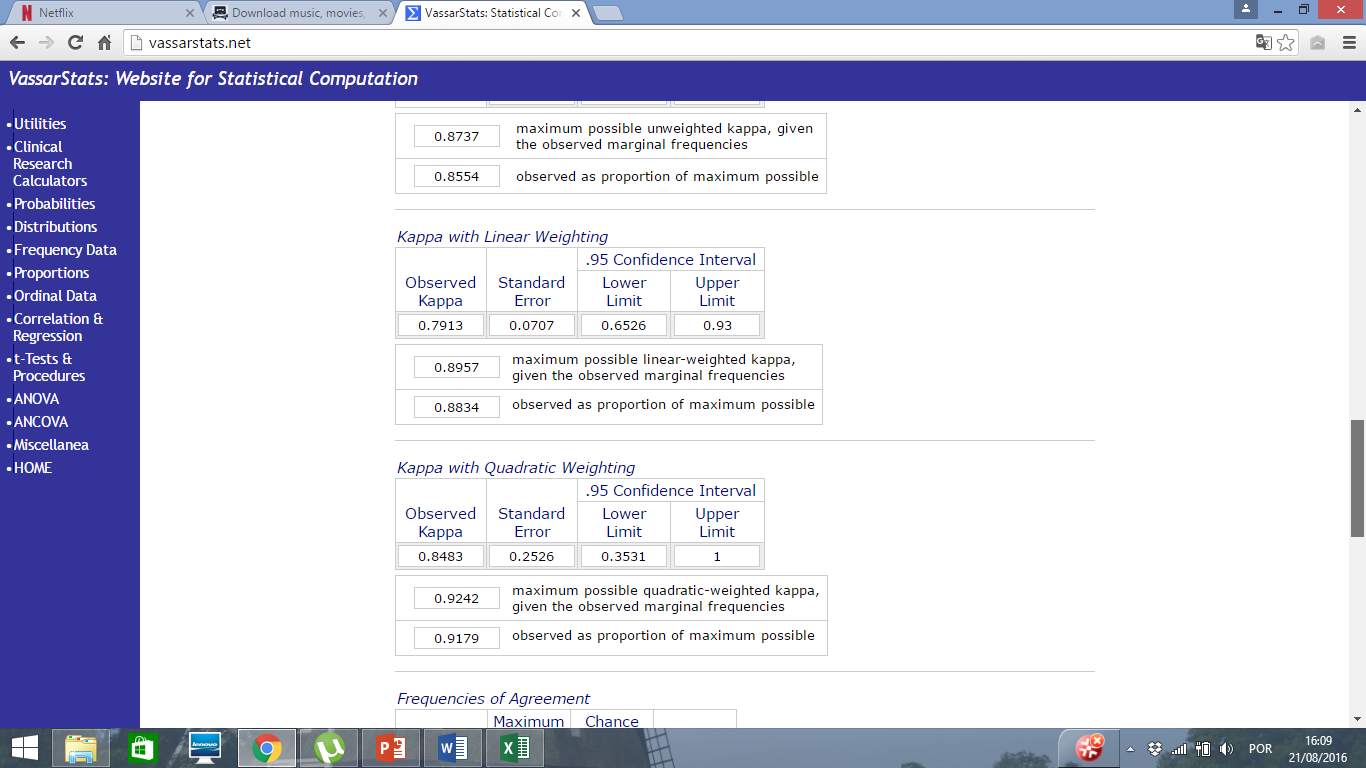


Scanner


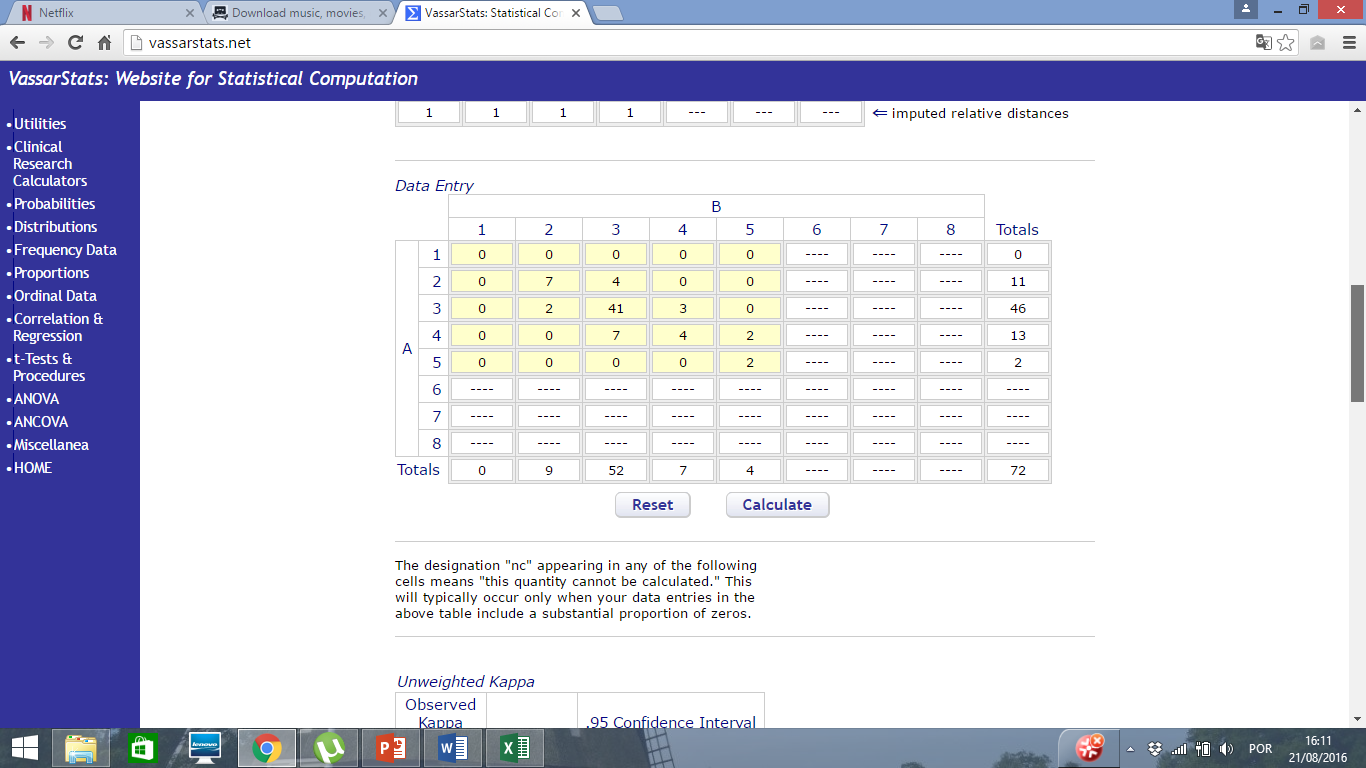


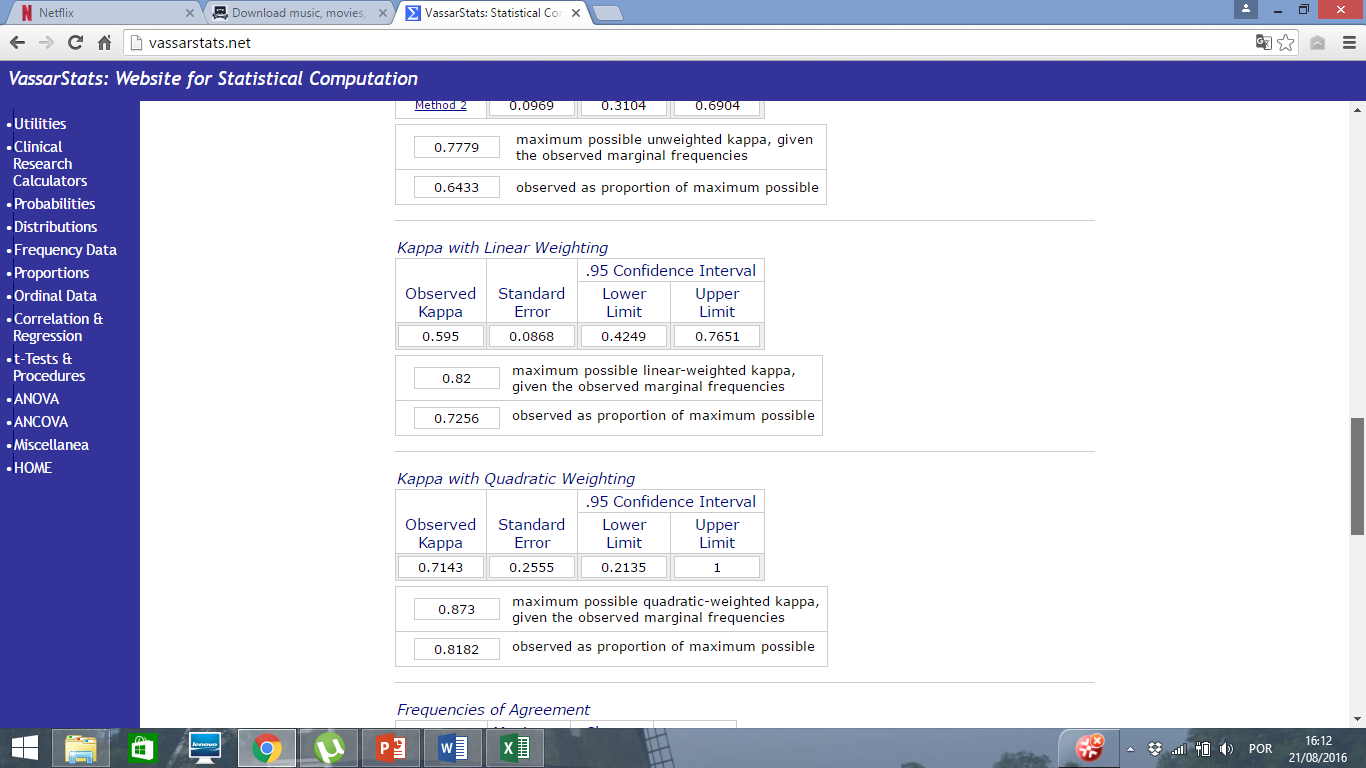


Friedman


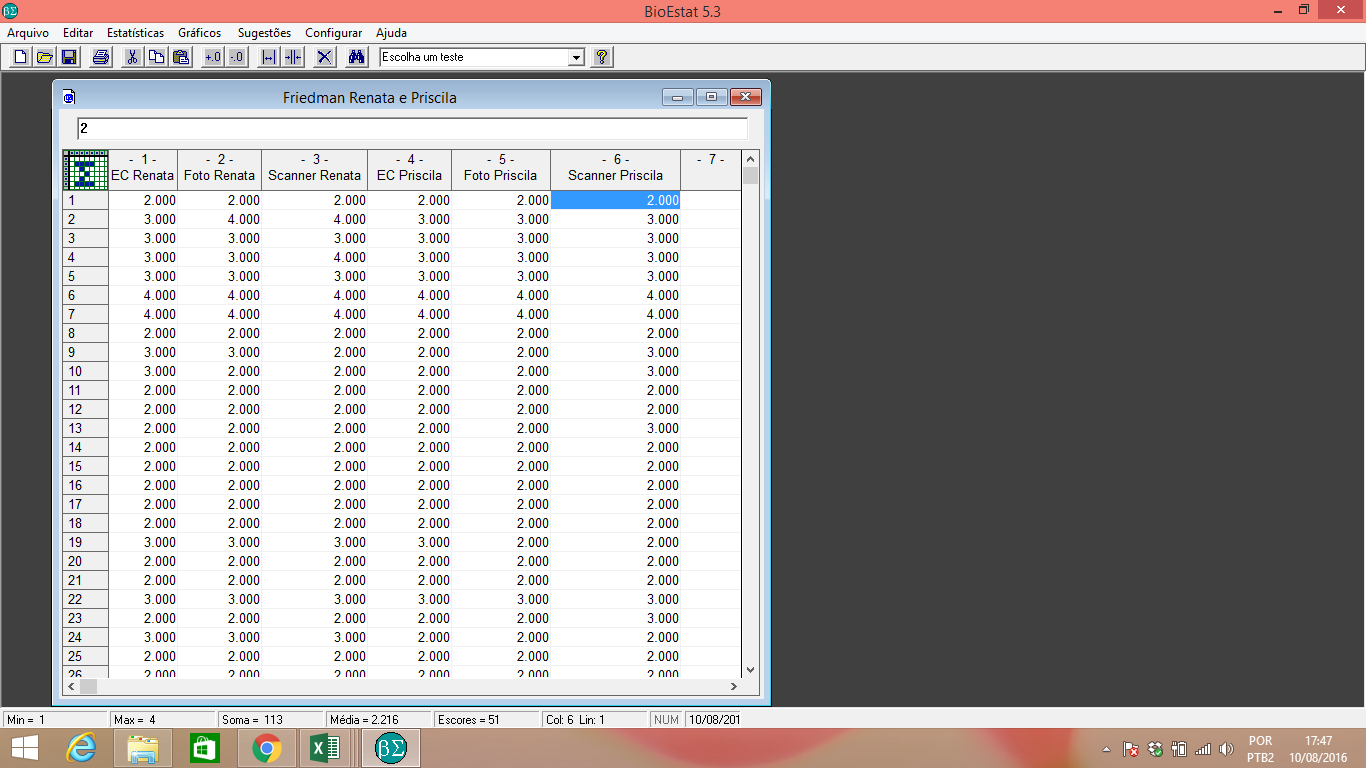


Friedman 1


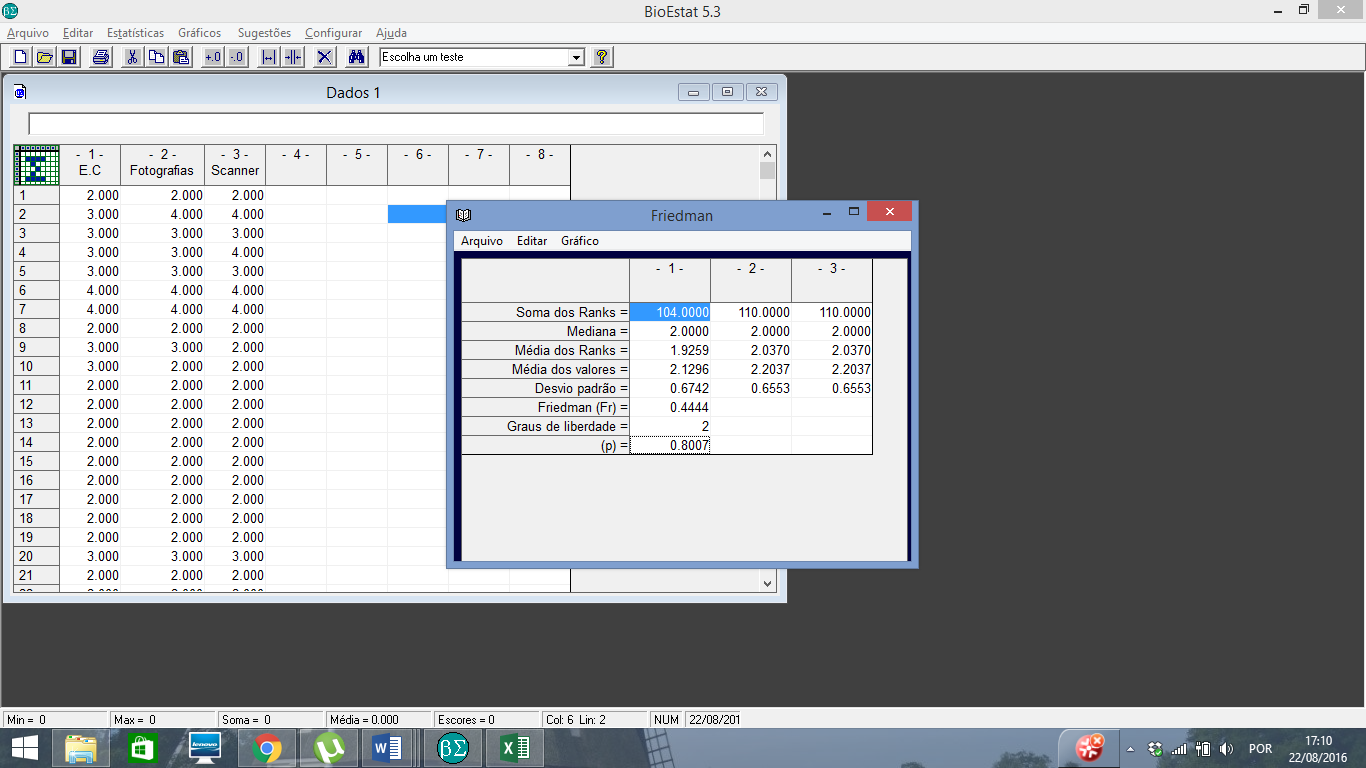


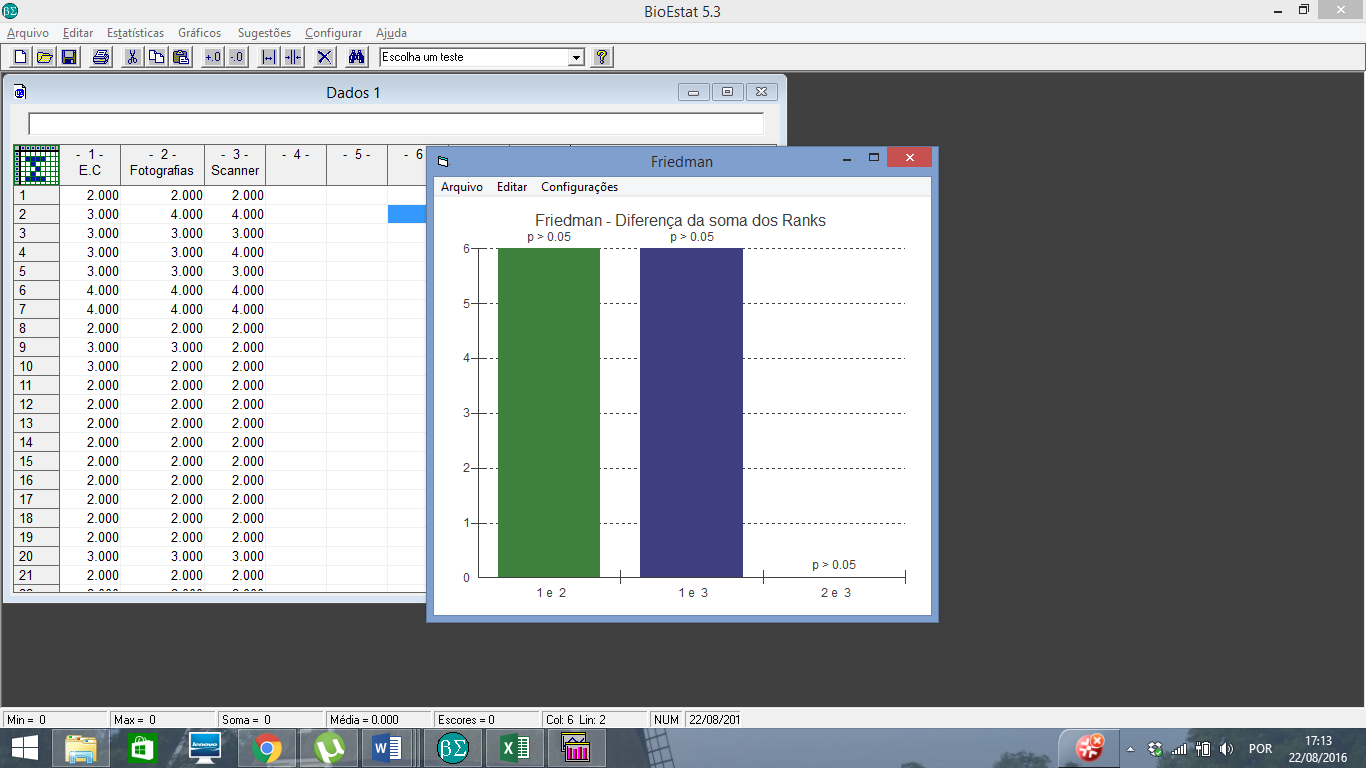


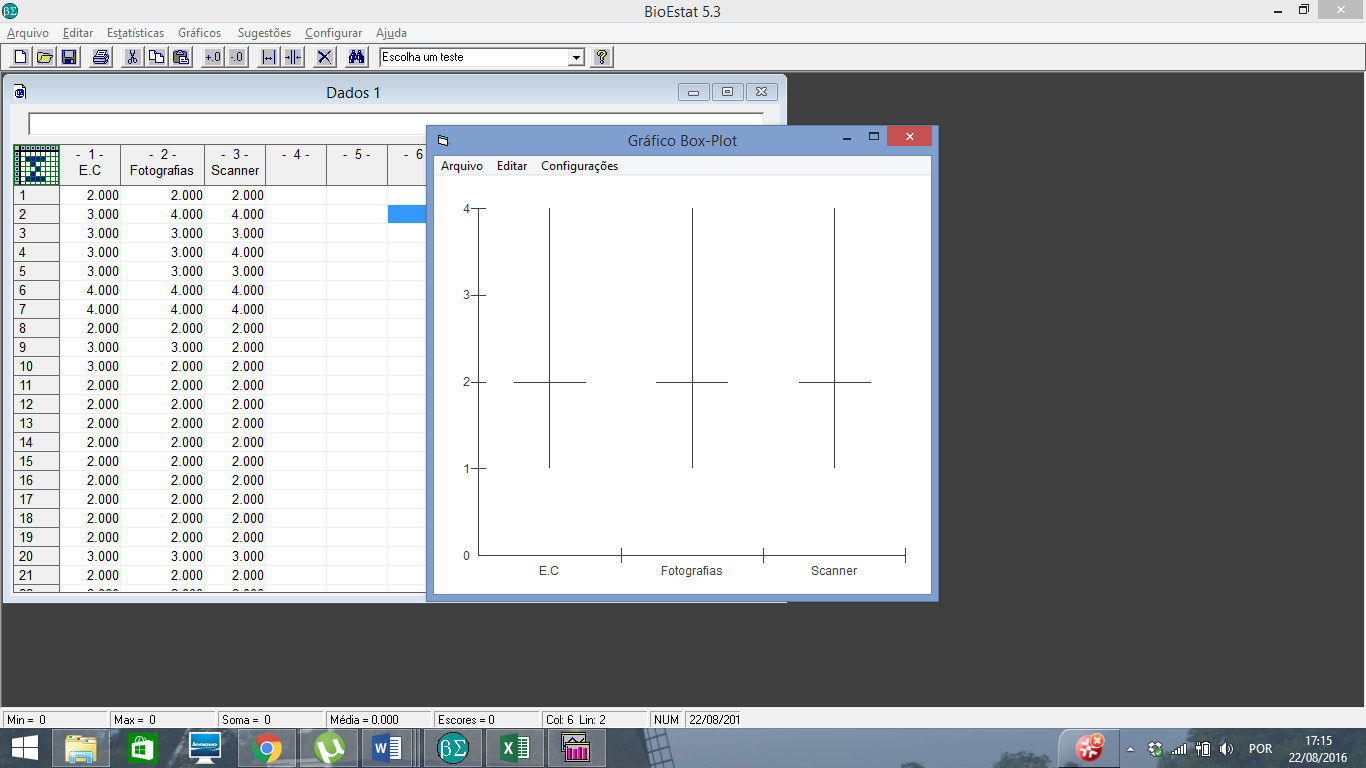


Friedman 2


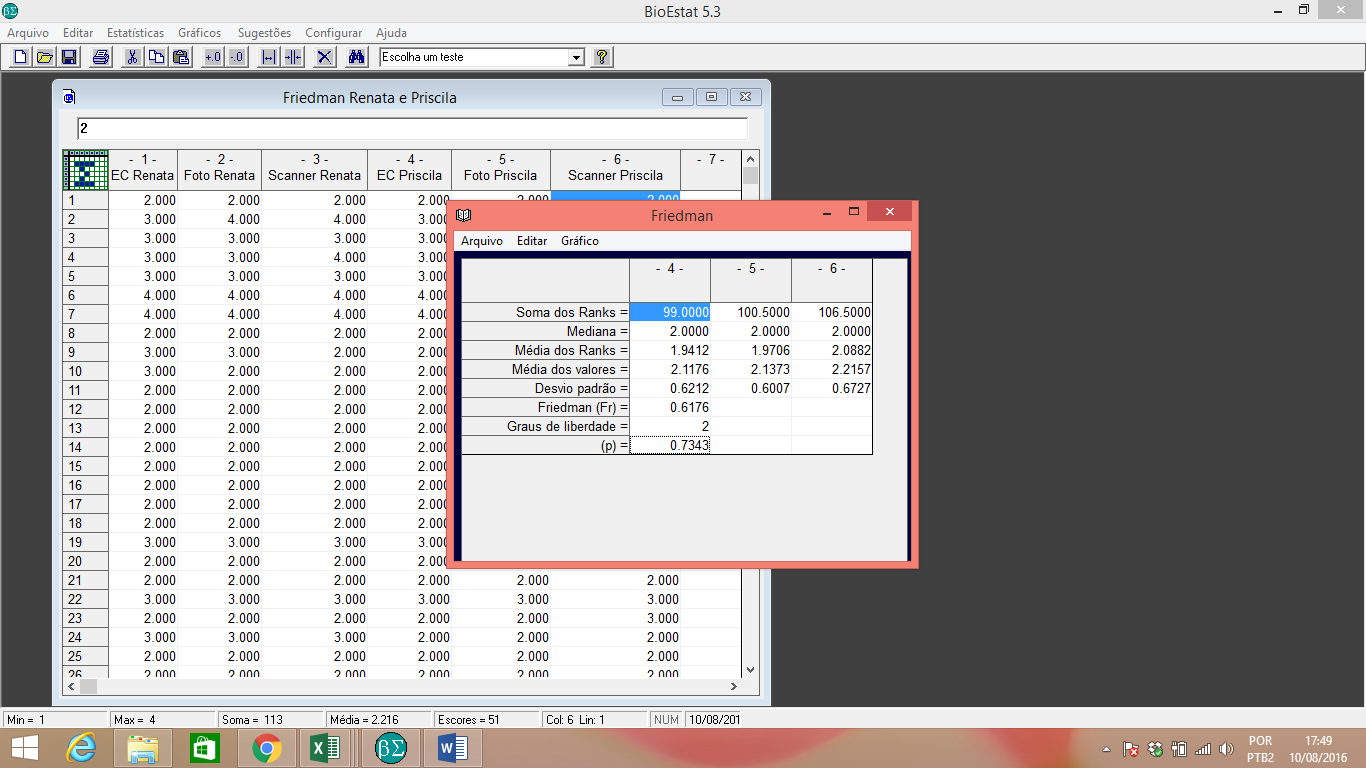


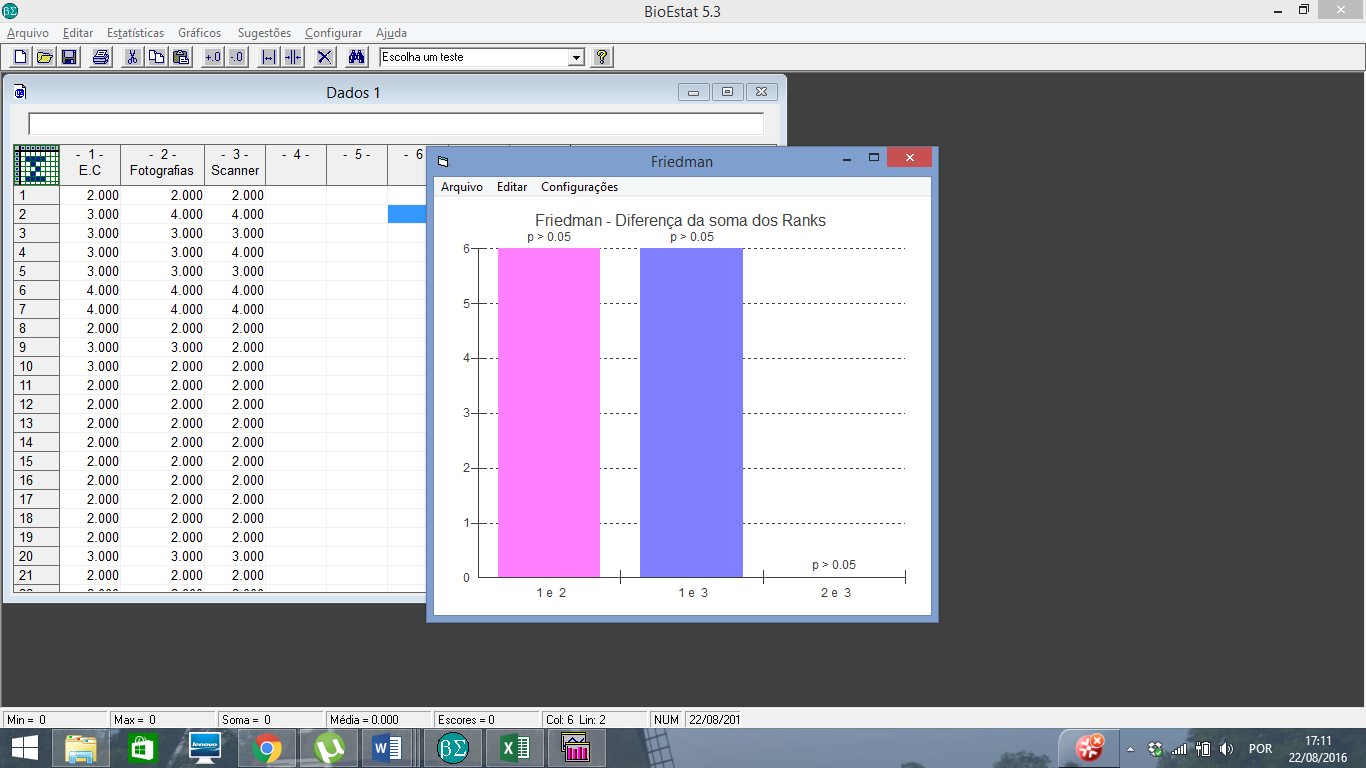


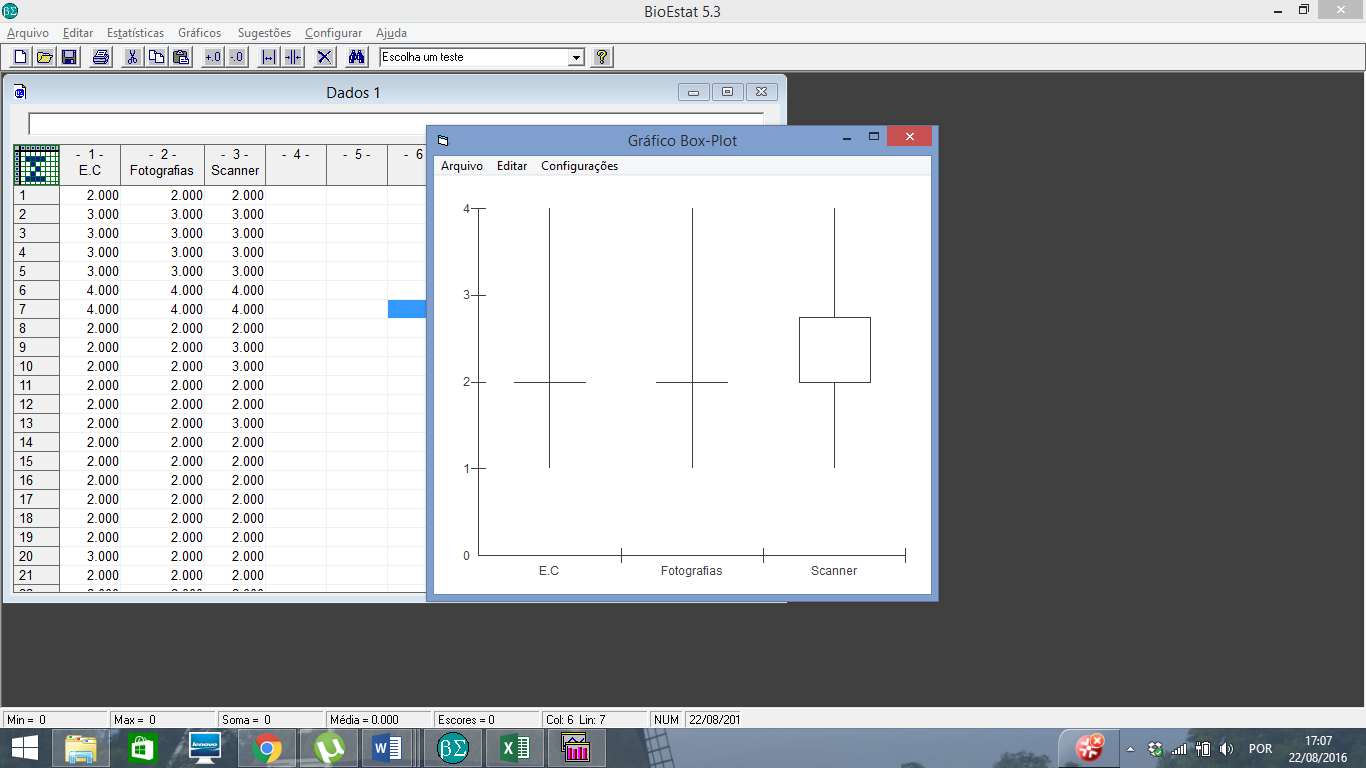


Bland-Altman


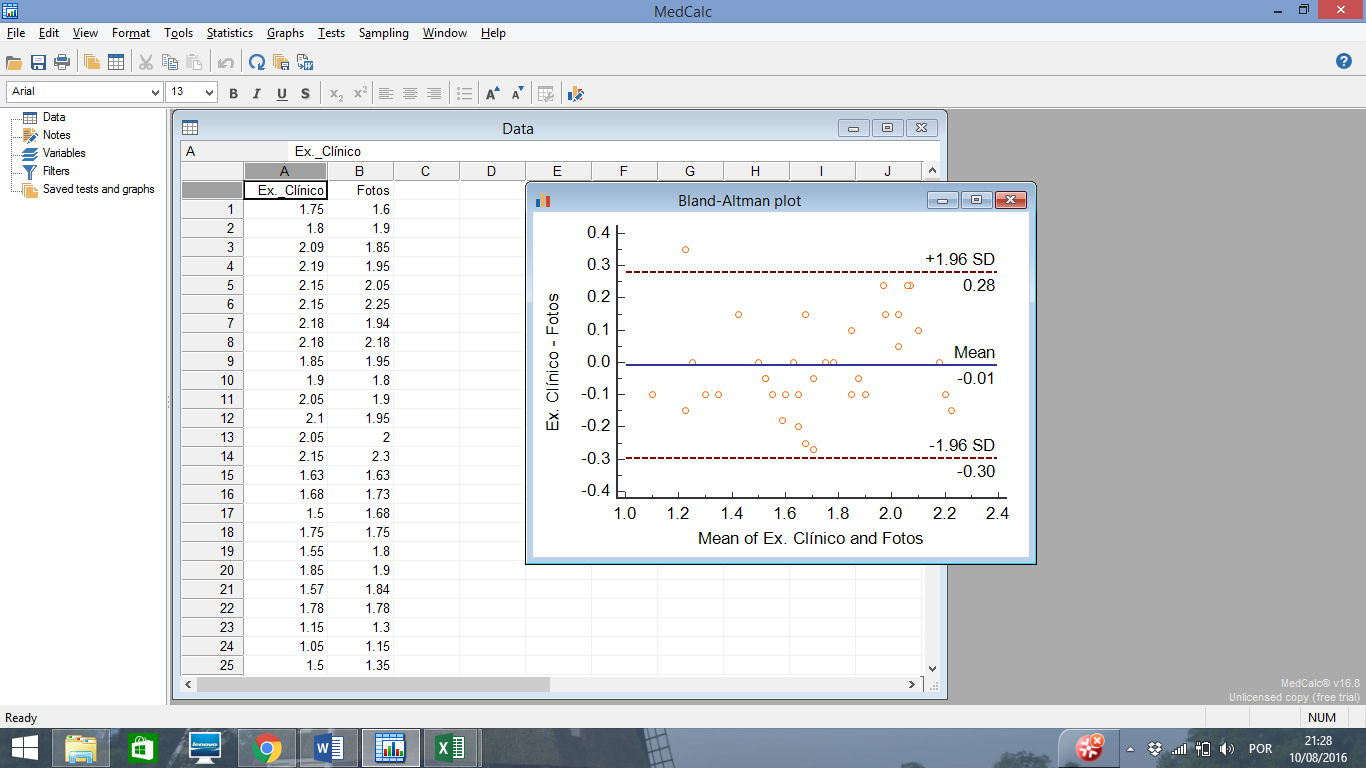


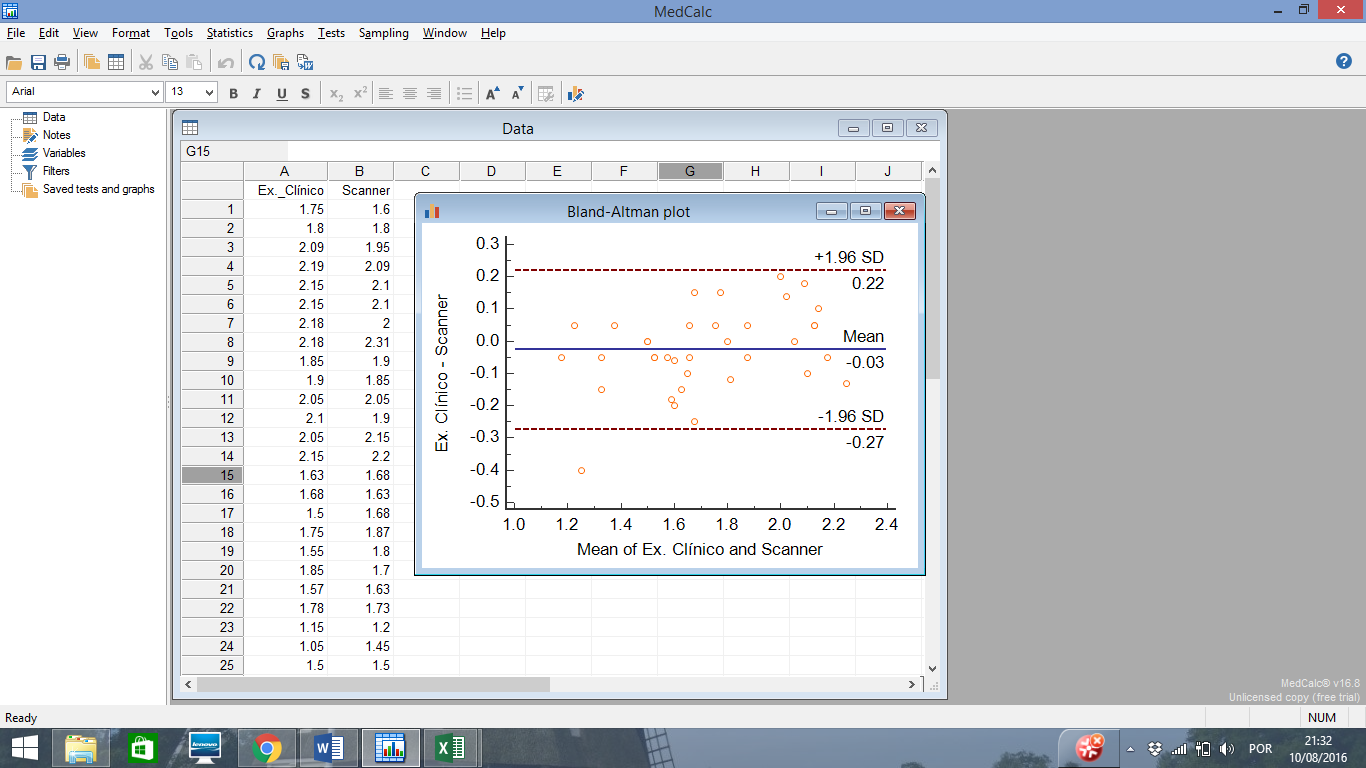


**Re evaluation**

Examiner 1


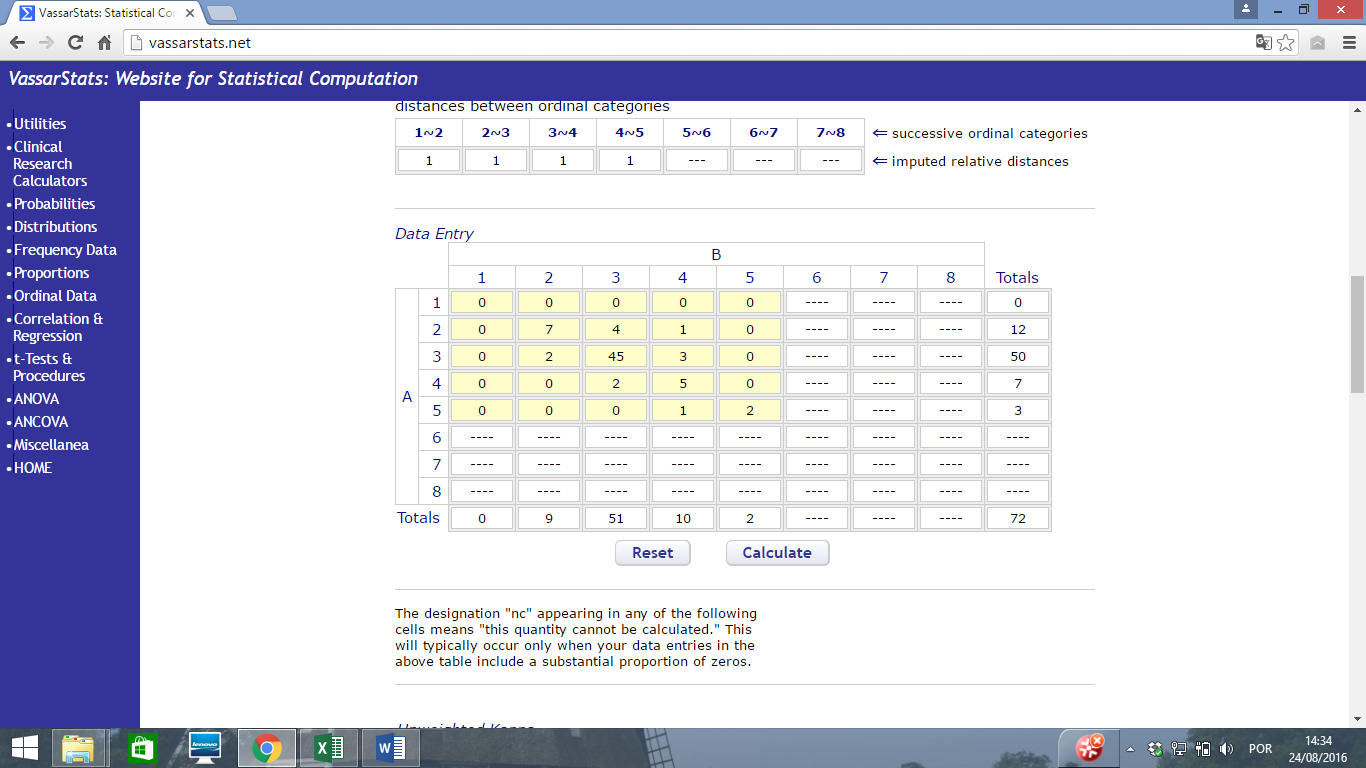


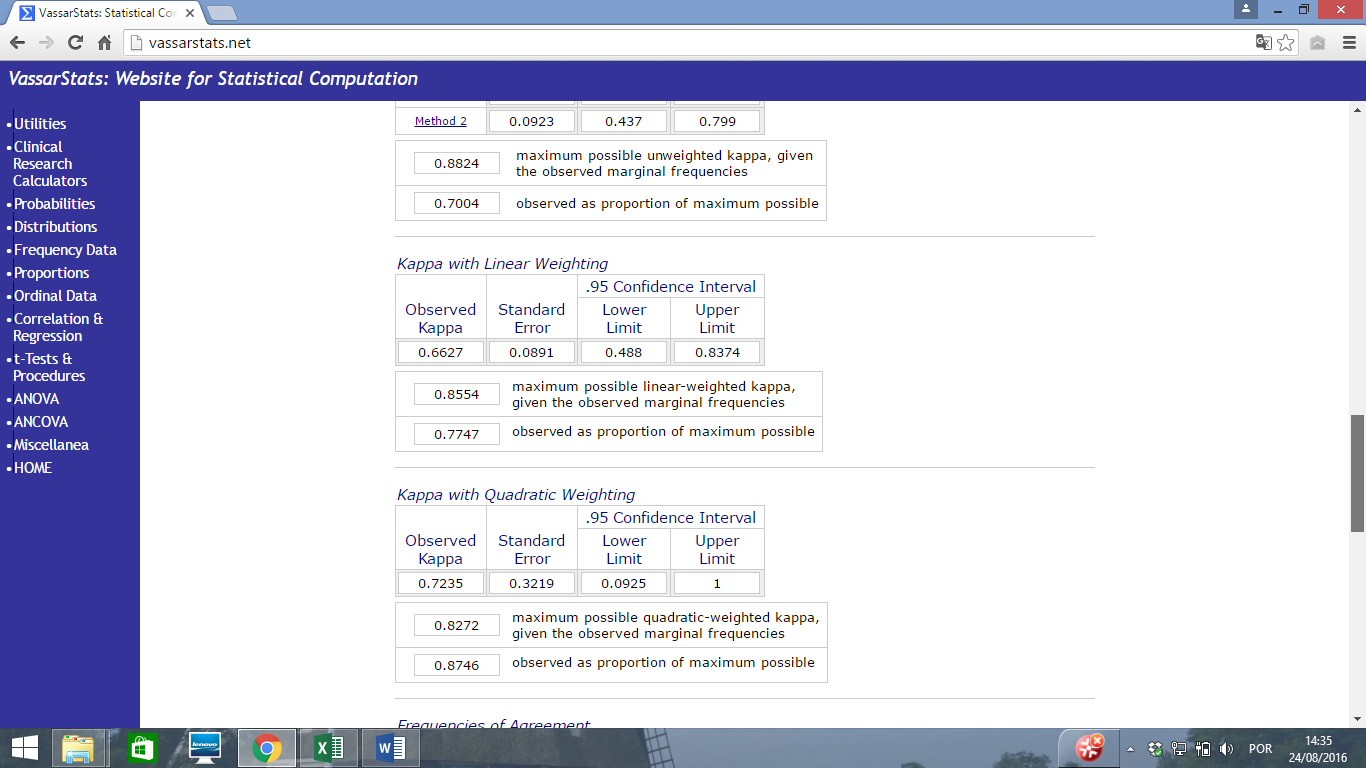


Examiner 2


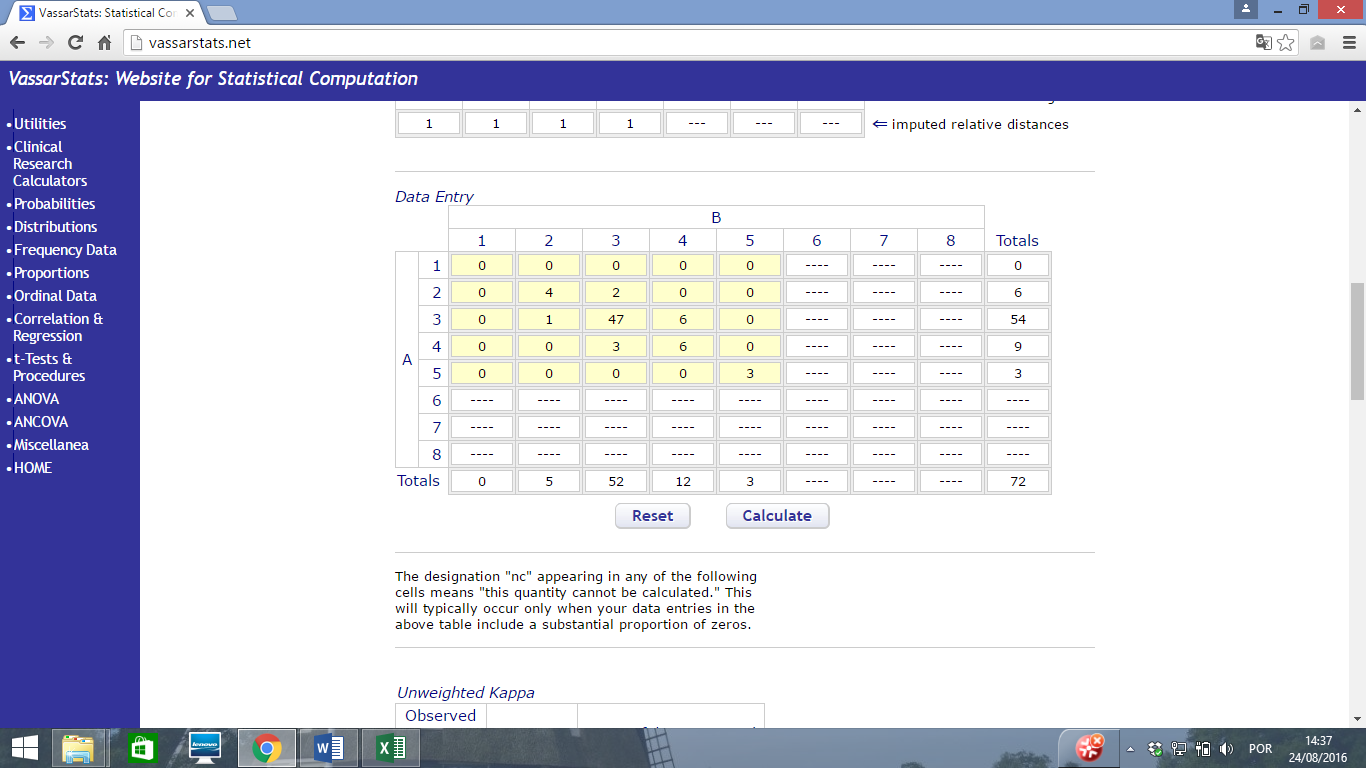


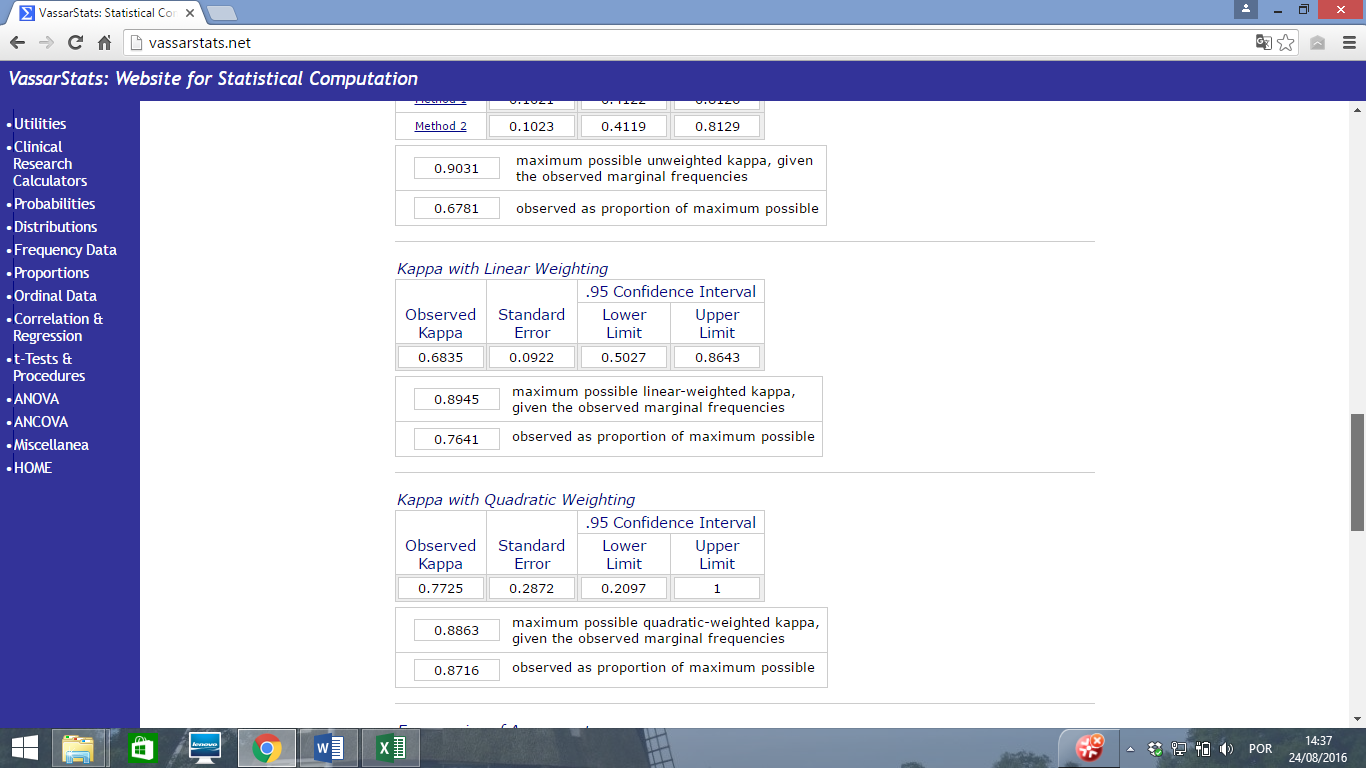

Supplement: S2 File — (DOCX) [file pone.0249119.s002.docx]
